# Multi-omics comparison of periodontitis and peri-implantitis identifies plasma cell enrichment as a shared feature and a periodontitis-associated endothelial–plasma cell APP–CD74 axis

**DOI:** 10.3389/fimmu.2026.1867656

**Published:** 2026-07-02

**Authors:** Xudong Tian, Xiaoxue Wang, Shensui Li, Mingyue Cui, Yang Chen, Ziqian Tian, Helin Chen, Xiaorong Fu

**Affiliations:** 1 Department of Oral and Maxillofacial Surgery, School of Stomatology & Hospital of Stomatology, Guizhou Medical University, Guiyang, Guizhou, China; 2 Department of Prosthetics and Implant, School of Stomatology & Hospital of Stomatology, Guizhou Medical University, Guiyang, Guizhou, China; 3 School of Biology and Engineering (School of Health Medicine Modern Industry), Guizhou Medical University, Guiyang, Guizhou, China

**Keywords:** APP-CD74, endothelial cells, peri-implantitis, periodontitis, plasma cells, single-cell RNA sequencing, spatial transcriptomics

## Abstract

**Objective:**

To define the shared and divergent immune architecture of periodontitis (PD) and peri-implantitis (PI) and to identify intercellular communication programs linked to plasma cell accumulation.

**Methods:**

Differentially expressed genes (DEGs) in PD and PI versus healthy controls were identified from two bulk datasets (GSE223924, GSE106090); robust cross-dataset common DEGs underwent functional enrichment and protein–protein interaction analysis. A single-cell atlas (GSE171213) supported dual deconvolution (BisqueRNA, CIBERSORTx) and CellChat-based, plasma cell-centered communication inference. Key findings were independently validated in two additional periodontitis bulk cohorts (GSE10334, GSE16134) and an independent periodontitis single-cell dataset (GSE164241). Candidate ligand–receptor interactions were evaluated by spatial transcriptomics (GSE206621) and multiplex immunofluorescence in a rat periodontitis model.

**Results:**

PI showed broader transcriptomic perturbation than PD (1,832 PI-specific and 820 PD-specific cross-validated DEGs). A total of 693 robust common DEGs with 99.0% directional concordance defined a shared inflammatory core enriched in cytokine signaling, B-cell activation, and osteoclast differentiation. Single-cell analysis resolved 15 cell populations and marked immune remodeling in periodontitis. Dual deconvolution identified plasma cell enrichment as the most reproducible infiltration feature across datasets and methods. CellChat revealed disease-associated remodeling of the plasma cell communication network, with endothelial cells as dominant senders and APP-CD74 as the strongest candidate incoming axis. Spatial transcriptomics showed a 14.43-fold increase in spot-level APP-CD74 co-enrichment in periodontitis-affected tissue, and multiplex immunofluorescence supported enhanced protein-level coupling between APP^+^CD31^+^ endothelial and CD74^+^CD138^+^ plasma cell domains.

**Conclusions:**

Plasma cell enrichment is the most robust shared immune feature of PD and PI. In periodontitis, convergent multi-omics and tissue-level evidence supports a candidate endothelial–plasma cell communication program centered on APP-CD74, suggesting that endothelial–plasma cell niche remodeling may sustain periodontal inflammation and inform host-modulatory strategies. Importantly, this axis was established only in periodontitis; its relevance to peri-implantitis remains inferential and will require direct single-cell and spatial validation in peri-implant tissue.

## 1 Introduction

Periodontitis is a chronic inflammatory disease of the tooth-supporting tissues initiated by dysbiotic biofilms and sustained by a dysregulated host response. It remains one of the most prevalent oral diseases worldwide, with severe periodontitis affecting approximately 1.1 billion people globally and contributing substantially to oral disability and tooth loss (1, 2). Peri-implantitis (PI), the implant-associated counterpart of periodontal breakdown, has emerged as a major biological complication of implant therapy, with contemporary meta-analyses estimating a patient-level prevalence of approximately 20% (95% CI ~13%–26%) (3, 4). Its clinical management remains challenging, as highlighted in the recent European Federation of Periodontology (EFP) S3-level clinical practice guideline, which underscores the persistent therapeutic difficulty of established peri-implantitis lesions (5). Although periodontitis (PD) and PI share the core clinical features of mucosal inflammation, progressive bone loss, and eventual failure of the affected tooth or implant unit, accumulating evidence indicates that PI lesions are often larger, more inflammatory, and clinically more difficult to control than PD lesions (5–8).

The pathogenesis of both diseases is now understood as a consequence of reciprocal interactions between the subgingival biofilm and the host immune-inflammatory network (9, 10). In PD, pathogen-associated ligands activate pattern-recognition pathways, including TLR2-dependent signaling, leading to NF-κB-driven cytokine production, leukocyte recruitment, osteoclast activation, and connective tissue destruction (11, 12). Advanced periodontal lesions are characterized by marked B-cell and plasma-cell accumulation together with sustained local immunoglobulin-related responses (13, 14). In PI, this inflammatory background may be further modified by implant-specific factors, including titanium particle release and altered foreign body-associated tissue responses, which have been implicated in macrophage activation, cytokine amplification, and microenvironmental remodeling (15, 16). Consistent with this view, classic histopathologic studies have shown that peri-implantitis lesions are larger than periodontitis lesions and exhibit abundant plasma cells, macrophages, and neutrophils, supporting the concept that the two conditions are related but not identical inflammatory entities (14). Among the infiltrating populations, plasma cells are particularly noteworthy: they are consistently abundant in both PD and PI lesions and are thought to sustain local humoral inflammatory activity, yet the signals that recruit and retain them within diseased gingival tissue remain poorly defined.

Recent transcriptomic studies have begun to define both the shared and distinct molecular features of PD and PI. Comparative bulk RNA profiling has shown broad overlap in inflammatory and immune-response genes, while also suggesting that PI may display stronger vascular, stromal, and innate immune activation signatures than PD (17, 18). However, bulk transcriptomics averages signals across heterogeneous cell populations and therefore cannot determine which cell compartments drive the observed molecular changes. This limitation is particularly relevant in plasma cell-rich lesions, in which shifts in cellular composition may substantially influence the apparent expression of inflammatory pathways.

Single-cell RNA sequencing (scRNA-seq) and computational tissue deconvolution now provide a framework to bridge this gap. Human gingival scRNA-seq studies have identified diverse epithelial, stromal, endothelial, and immune populations and have revealed inflammation-associated expansion of specific myeloid and fibroblast subsets in periodontitis (19, 20). In parallel, reference-based deconvolution approaches such as BisqueRNA and digital cytometry tools such as CIBERSORTx have enabled estimation of cell-type composition from bulk tissue transcriptomes using single-cell-informed or immune reference matrices (21, 22). These methods are particularly valuable for comparative studies of PD and PI, where direct single-cell datasets remain limited, especially for peri-implantitis.

Beyond cellular composition, an important unresolved question is how immune-rich periodontal lesions are spatially organized and maintained. Cell–cell communication inference frameworks such as CellChat enable systematic prediction of ligand–receptor interactions from scRNA-seq data and can uncover candidate signaling programs that are not evident from differential expression alone (23, 24). Likewise, spatial transcriptomics has begun to define the topographic organization of periodontal tissues, revealing site-restricted inflammatory niches and structured stromal–immune interactions in diseased gingiva (25, 26). Yet despite the recognized prominence of plasma cells in advanced periodontal lesions, the vascular and stromal signals that recruit and retain these cells in diseased tissue remain poorly defined at cellular resolution. This gap is particularly relevant because plasma-cell-rich infiltrates are a shared histopathologic feature of PD and PI, but the mechanisms sustaining these niches have not been systematically dissected by converging molecular, cellular, spatial, and tissue-level evidence (6, 14).

In the present study, we used an integrative multi-omics strategy combining cross-platform bulk transcriptomics, scRNA-seq-based cellular mapping, dual deconvolution analysis, plasma cell-centered CellChat inference, spatial transcriptomic validation, and tissue-level multiplex immunofluorescence. We aimed to define the shared and divergent inflammatory architecture of PD and PI while identifying disease-associated intercellular communication programs linked to plasma cell accumulation. Our analyses revealed a robust shared inflammatory core between PD and PI, identified plasma cell enrichment as the most reproducible cellular feature across datasets and deconvolution frameworks, and provided convergent evidence in periodontitis for a disease-associated endothelial–plasma cell communication program centered on APP–CD74. Together, these findings nominate endothelial–plasma cell niche remodeling as a testable mechanism in destructive periodontal inflammation and provide a framework for future mechanistic and translational studies. Beyond mechanistic insight, delineating this vascular–immune interface may provide new entry points for host-modulatory strategies in patients with persistent, destructive periodontal lesions.

## 2 Materials and methods

### 2.1 Study design and public datasets

This study used a multi-layer integrative design combining bulk transcriptomics, single-cell RNA sequencing (scRNA-seq), cell-type deconvolution, cell-cell communication analysis, spatial transcriptomics, and tissue-level multiplex immunofluorescence validation. Publicly available human transcriptomic datasets were retrieved from the Gene Expression Omnibus (GEO) database. Because all human omics data analyzed in this study were obtained from publicly accessible and de-identified repositories, additional institutional approval for human subjects was not required.

Two bulk transcriptomic datasets were included for comparative analysis of healthy gingiva, periodontitis (PD), and peri-implantitis (PI). GSE223924 is an RNA-sequencing dataset generated on platform GPL24676 and contains 30 human gingival tissue samples, with 10 samples each from healthy controls, PD, and PI. GSE106090 is a microarray dataset generated on platform GPL21827 and contains 18 gingival tissue samples, with six samples per group. For scRNA-seq reference construction and downstream cell-cell communication analysis, GSE171213 was used. Because the present study focused on active disease versus healthy states, only healthy and periodontitis samples were retained, whereas post-treatment samples were excluded. For spatial validation, GSE206621, a human oral mucosa spatial transcriptomic dataset including healthy and periodontitis-affected individuals, was analyzed. This dataset was used to assess whether candidate plasma cell-associated communication signals inferred from periodontitis scRNA-seq data showed spatial support in periodontitis-affected oral mucosa. Because no peri-implantitis spatial transcriptomic dataset was publicly available, GSE206621 provided spatial support for periodontitis only; periodontitis-affected oral mucosa samples were treated as the disease group and compared with healthy oral mucosa, and no inference regarding peri-implant tissue was drawn from this dataset.

In addition, two independent periodontitis bulk transcriptomic datasets, GSE10334 and GSE16134, were used for external validation of candidate gene expression and inferred cellular composition. After sample filtering and dataset integration, these two cohorts included 557 gingival tissue samples in total, comprising 133 healthy and 424 periodontitis samples. The independent scRNA-seq dataset GSE164241 was further analyzed as an external single-cell validation cohort after excluding buccal mucosa samples; after quality control, doublet removal, and cell-type annotation, 21 samples comprising 81,975 annotated cells were retained for downstream cell-type composition and communication analyses.

### 2.2 Bulk transcriptomic analysis

#### 2.2.1 Data preprocessing

Bulk transcriptomic analyses were performed independently for GSE223924 and GSE106090 in R. For GSE223924, log-counts per million-normalized expression values were used directly. For GSE106090, probe-level expression data were matched to gene annotations and collapsed to gene symbols before downstream analysis. Expression matrices were aligned with sample metadata and restricted to samples annotated as healthy, PD, or PI.

#### 2.2.2 Differential expression analysis

Differential expression analysis was conducted using the limma framework. A no-intercept design matrix was fitted for each dataset, and two primary contrasts were evaluated: PI versus healthy and PD versus healthy. Linear models were fitted and followed by empirical Bayes moderation. Differentially expressed genes (DEGs) were defined using an absolute log2 fold-change greater than 1 and a Benjamini-Hochberg adjusted P value below 0.05. Volcano plots were generated with ggplot2, and selected genes were labeled with ggrepel. Heatmaps were constructed using row-scaled expression values and Euclidean distance-based clustering.

#### 2.2.3 Cross-dataset integration and robust DEG definition

To identify reproducible signals across platforms, within-dataset intersections were first calculated between PI-versus-healthy and PD-versus-healthy DEG sets. Cross-dataset intersections were then computed between GSE106090 and GSE223924. Robust common DEGs were defined as genes differentially expressed in both disease comparisons in both datasets. Directional reproducibility was assessed by comparing the sign of fold change across datasets. Disease-specific DEGs were defined as genes reproducibly validated across both datasets exclusively in one comparison.

#### 2.2.4 Functional enrichment analysis

Functional annotation was performed for robust common DEGs as well as disease-specific DEG sets. Gene symbols were converted to Entrez identifiers using clusterProfiler with org.Hs.eg.db as the annotation database. Gene Ontology enrichment analysis was performed for biological process, cellular component, and molecular function categories. KEGG and Reactome pathway enrichment analyses were then conducted. In all enrichment analyses, Benjamini-Hochberg correction was applied, and adjusted P values and q values below 0.05 were considered significant.

#### 2.2.5 Protein-protein interaction network analysis

To characterize the interaction architecture of robust common DEGs, a protein-protein interaction network was constructed using the STRING database with a minimum interaction confidence score of 0.4. The resulting network was imported into R for graph-based analysis. Hub genes were ranked using a consensus strategy integrating degree, betweenness, closeness, and eigenvector centrality. The top 10 genes were defined as hub genes. An extended core network was subsequently generated by extracting hub genes and their first-degree neighbors and was visualized using igraph-, tidygraph-, and ggraph-based workflows.

### 2.3 Single-cell RNA-seq analysis

#### 2.3.1 Data processing and quality control

The scRNA-seq dataset GSE171213 was analyzed using Seurat. From the original cohort, four healthy and five severe periodontitis samples were retained, whereas three post-treatment samples were excluded. Raw count matrices were imported on a per-sample basis and subjected to quality control. Cells were retained if the number of detected genes and total UMI counts fell between 80 and the sample-specific 99th percentile, and if the mitochondrial transcript fraction was below 40%. Potential doublets were identified for each sample using scDblFinder and removed before integration.

#### 2.3.2 Normalization, dimensional reduction, and batch correction

After quality control, data were log-normalized, and the top 2,000 highly variable genes were identified using the variance-stabilizing transformation method. Scaled expression matrices were generated with regression of mitochondrial gene percentage. Principal component analysis was performed, and Harmony was applied to correct sample-level batch effects. Uniform manifold approximation and projection embedding was then computed using Harmony-corrected dimensions. Graph-based clustering was performed using the Louvain algorithm, and cluster stability across resolutions was evaluated using clustree. A resolution of 0.7 was selected for downstream analysis.

#### 2.3.3 Cell-type annotation

Cluster marker genes were identified using the Wilcoxon rank-sum test. Cell-type identities were assigned according to established lineage markers, including KRT5 and KRT14 for epithelial cells, PDGFRA and COL1A1 for fibroblasts, PECAM1 and VWF for endothelial cells, RGS5 for pericytes, CD3E and CD4 for CD4+ T cells, CD3E and CD8A for CD8+ T cells, MS4A1 for B cells, MZB1 for plasma cells, NKG7 and GZMA for NK cells, FCGR3B and CXCR2 for neutrophils, CD163 for macrophages, TPSAB1 for mast cells, LILRA4 for plasmacytoid dendritic cells, CLEC9A for cDC1, and MKI67 for cycling cells. Two artifact clusters were removed, and redundant clusters were merged, yielding 15 final annotated cell populations.

### 2.4 Cell-type deconvolution of bulk transcriptomes

To infer the cellular composition of bulk gingival transcriptomes, two complementary deconvolution strategies were applied.

First, reference-based deconvolution was performed using BisqueRNA. The cleaned and annotated GSE171213 scRNA-seq dataset served as the reference, with expression and metadata assembled into an ExpressionSet object. Bulk matrices from GSE106090 and GSE223924 were formatted in the same framework, and cell-type proportions were estimated using ReferenceBasedDecomposition. Estimated proportions were normalized to sum to one within each sample.

Second, orthogonal immune deconvolution was performed using CIBERSORTx with the LM22 immune cell signature matrix in absolute mode and 1,000 permutations. For this analysis, bulk expression matrices were back-transformed from log-transformed values to linear CPM space before upload. CIBERSORTx output tables were inspected for valid deconvolution results before downstream visualization. For heatmap or summary visualization, LM22 immune populations with all-zero or near-zero inferred abundance were omitted to avoid displaying non-informative cell types; specifically, cell types with a mean inferred proportion ≤ 0.005 across the visualized samples were excluded from the corresponding visualization panel. Therefore, the number of displayed CIBERSORTx cell types may differ across datasets or figures depending on dataset-specific abundance profiles, whereas the complete deconvolution output was retained for statistical evaluation unless otherwise specified.

For both methods, differences in inferred cell proportions among healthy, PD, and PI groups were assessed using the Kruskal-Wallis test, followed by pairwise Wilcoxon rank-sum tests with Benjamini-Hochberg correction when appropriate. Cell types with an adjusted P value below 0.05 were considered significantly altered. Cross-dataset reproducibility was evaluated by comparing -log10 adjusted P values between GSE106090 and GSE223924.

To further assess the robustness of plasma cell enrichment inferred from bulk transcriptomic data, plasma cell proportions estimated by BisqueRNA and CIBERSORTx were harmonized at the sample level across GSE106090 and GSE223924. The plasma cell columns from the two methods were standardized and merged by sample identifier and dataset. Spearman correlation analysis was performed across matched samples to evaluate sample-level concordance between the two deconvolution frameworks. To assess directional consistency across clinical groups, matched estimates from both datasets were combined, and group-wise plasma cell proportions were summarized as mean ± SE for each method. Group comparisons within each dataset and method were assessed using Kruskal–Wallis tests followed by Bonferroni-adjusted Dunn’s *post hoc* tests where applicable.

### 2.5 Cell-cell communication analysis

To investigate disease-associated intercellular communication networks, CellChat was applied to the cleaned scRNA-seq dataset after stratification into healthy and periodontitis groups. Separate CellChat objects were generated from normalized expression matrices using Seurat-derived cell annotations. The human CellChat ligand-receptor database was used as the interaction reference.

Overexpressed genes and ligand-receptor pairs were identified using the default CellChat workflow. Communication probabilities were computed with population.size = TRUE, and interactions involving cell populations with fewer than 10 cells were excluded. Pathway-level communication probabilities were then summarized using standard CellChat aggregation functions. Because plasma cells emerged as a key disease-associated compartment in earlier analyses, plasma cell-centered networks were examined in both sender and receiver modes. For receiver-mode analysis, the number of significant ligand-receptor interactions and the summed communication probability from each source cell population to Plasma Cells were quantified and compared between healthy and periodontitis groups. In addition, endothelial-to-plasma cell ligand-receptor interactions were extracted separately to characterize vascular niche-associated signaling, with APP-CD74 highlighted as a candidate disease-associated communication axis. Both the number of inferred interactions and the summed communication probability were therefore reported for each source-to-Plasma-Cell channel, whereas individual ligand-receptor pairs (including APP-CD74) were ranked by their CellChat communication probability; unless otherwise stated, statements describing a signal as “dominant” or “strongest” refer to communication probability rather than interaction count. Statistical significance was evaluated by permutation testing, with *P* < 0.05 considered significant and *P* < 0.01 interpreted as high-confidence communication.

### 2.6 Validation analyses using original and independent periodontitis cohorts

To further evaluate the robustness of the candidate plasma cell-endothelial inflammatory program, targeted validation analyses were performed using both the original bulk datasets and independent periodontitis cohorts. A predefined 32-gene panel was examined, including robust common hub genes, plasma cell markers, plasma cell-associated receptor genes, endothelial markers, and endothelial-associated ligand or extracellular matrix genes. For GSE106090 and GSE223924, available target genes were extracted and visualized as row-scaled heatmaps across healthy, PD, and PI groups. Because gene coverage differed across platforms, only genes detected after dataset-specific annotation were retained for visualization and statistical testing. Accordingly, 29 of 32 target genes were available in GSE106090, 32 of 32 in GSE223924, and 30 of 32 in the integrated GSE10334/GSE16134 validation cohort. Group differences were assessed using the Kruskal-Wallis test followed by Bonferroni-adjusted Dunn’s *post hoc* test where applicable.

For external bulk validation, GSE10334 and GSE16134 were integrated after normalization, probe-to-gene annotation, and batch-effect correction. GSE10334 contained 247 samples, including 64 healthy and 183 periodontitis samples, whereas GSE16134 contained 310 samples, including 69 healthy and 241 periodontitis samples, yielding a combined validation cohort of 557 gingival tissue samples. Differential expression of available target genes between healthy and periodontitis samples was assessed and visualized using row-scaled heatmaps with group and dataset annotations.

To validate cell-type alterations in independent bulk cohorts, GSE10334 and GSE16134 were analyzed using both CIBERSORTx and BisqueRNA. CIBERSORTx was performed using the LM22 immune signature matrix, whereas BisqueRNA used the annotated GSE171213 scRNA-seq dataset as the reference. For CIBERSORTx-based visualization, only samples with valid deconvolution results were retained according to the CIBERSORTx output P value, and low-abundance LM22 cell types with mean inferred proportions ≤0.005 were omitted from heatmap visualization to avoid displaying all-zero or near-zero populations. Therefore, the number of visualized CIBERSORTx cell types may differ across panels depending on dataset-specific abundance profiles, while the complete deconvolution output was retained for statistical evaluation. Cell-type differences between healthy and periodontitis samples were evaluated with multiple-testing correction.

For independent single-cell validation, GSE164241 was analyzed after excluding buccal mucosa samples. The remaining samples were processed using a Seurat-based workflow, including quality control, doublet removal, normalization, dimensional reduction, clustering, and broad cell-type annotation. After these preprocessing and annotation steps, the final validation dataset contained 21 samples, including 13 healthy and 8 periodontitis samples, and 81,975 quality-controlled, doublet-filtered, and annotated cells, comprising 49,800 cells from healthy samples and 32,175 cells from periodontitis samples. Sample-level cell-type proportions were then compared between healthy and periodontitis groups. CellChat was applied separately to healthy and periodontitis samples to determine whether plasma cell-centered communication changes observed in GSE171213 could be reproduced in an independent scRNA-seq cohort. Plasma Cells were analyzed in both sender and receiver modes, and communication changes were calculated as periodontitis minus healthy.

### 2.7 Spatial transcriptomic analysis

Spatial transcriptomic validation was performed using GSE206621, which contains eight 10x Genomics Visium sections from healthy and periodontitis-affected adult oral mucosa. Data were processed in Seurat using the SCTransform normalization workflow. Plasma cell enrichment was quantified by module scoring with canonical plasma cell markers, including SDC1, MZB1, JCHAIN, IGHA1, IGHG1, XBP1, and PRDM1. Endothelial enrichment was scored using PECAM1, VWF, CDH5, CD34, and ENG.

To assess spatial support for candidate ligand-receptor axes, two complementary sample-level metrics were calculated for each gene pair: the Spearman correlation of normalized spot-level expression across each section, and the double-positive spot rate, defined as the proportion of spots in which both genes exceeded the 75th percentile within that section. Group comparisons between healthy and periodontitis-affected samples were performed using the Wilcoxon rank-sum test. Effect size was estimated using Cohen’s d. In addition, pooled spot-level correlation analyses and spatial visualization of co-expression or proximity patterns were used to further evaluate the APP-CD74 axis and endothelial-plasma cell spatial organization. All available sections were included in sample-level statistical analyses, whereas representative sections with adequate tissue coverage and visualization quality were selected for spatial feature plots. Because spot-level Visium data do not directly prove ligand-receptor binding or single-cell-level directionality, these analyses were interpreted as spatial support for candidate communication axes rather than direct functional validation.

### 2.8 Animal experiments

#### 2.8.1 Rat periodontitis model

To validate the inferred endothelial–plasma cell signaling axis at the protein level, an experimental periodontitis model was established in male Sprague–Dawley rats. A total of 18 eight-week-old male animals were randomly allocated, using a computer-generated random number sequence, into two groups of 9 animals each: a healthy control group and a periodontitis group. Group sizes were determined based on prior histological studies of ligature-induced rat periodontitis and to provide sufficient replicates for downstream multiplex immunofluorescence quantification.

For ligature placement, rats in the periodontitis group were anesthetized with 3% sodium pentobarbital solution at 40 mg/kg by intraperitoneal injection. Adequate anesthetic depth was confirmed by the absence of the pedal withdrawal reflex before the procedure. During anesthesia, animals were maintained under close observation for respiratory pattern and general condition. Periodontitis was induced by placing 4–0 silk ligatures around the cervical region of the bilateral maxillary second molars to promote plaque retention and periodontal inflammation. Ligatures were maintained for four weeks. Control animals received no ligature placement or other local intervention and were euthanized at the same experimental endpoint as the periodontitis group.

Animals were monitored during the experimental period for general health status, food intake, body weight change, ligature retention, and signs of distress. All animal procedures, including anesthesia and euthanasia, were approved by the Animal Experimental Ethics Inspection Committee of Guizhou Medical University (Approval No. 2403313).

#### 2.8.2 Tissue collection, fixation, decalcification, and paraffin sectioning

At the end of the four-week experimental period, rats were euthanized by overdose of 3% sodium pentobarbital solution at 150 mg/kg via intraperitoneal injection. Death was confirmed by cessation of respiration and heartbeat, together with the absence of corneal and pedal withdrawal reflexes. After confirmation of death, maxillary tissue blocks containing teeth and alveolar bone were harvested and fixed in 4% paraformaldehyde for 48 h.

Samples were decalcified in 10% EDTA (pH 7.4) at 4 °C for 4–6 weeks, with the solution replaced every 2–3 days. After complete decalcification, tissues were dehydrated through graded ethanol, cleared in xylene, embedded in paraffin, and sectioned longitudinally at a thickness of 5 μm. Sections were baked overnight at 60 °C before staining.

### 2.9 Multiplex immunofluorescence and image quantification

#### 2.9.1 Multiplex immunofluorescence staining

Multiplex immunofluorescence staining was performed using a tyramide signal amplification (TSA)-based sequential staining workflow to detect CD31, APP, CD74, and CD138, with DAPI used as the nuclear counterstain. Briefly, rat paraffin-embedded tissue sections were deparaffinized in xylene, rehydrated through graded ethanol, and subjected to heat-induced antigen retrieval in citrate buffer (pH 6.0). Endogenous peroxidase activity was quenched with 3% hydrogen peroxide, and nonspecific binding was blocked with 3% bovine serum albumin.

Sequential staining cycles were then performed. In each cycle, sections were incubated with one primary antibody overnight at 4 °C, followed by incubation with HRP-conjugated goat anti-rabbit IgG and development with the corresponding TSA fluorophore. After each staining round, bound primary and secondary antibody complexes were removed by microwave-mediated stripping before the next marker was applied, while the covalently deposited TSA signal was retained. The staining order was CD31, APP, CD74, and CD138. Primary antibodies were as follows: anti-CD31 (Abcam, Cat# ab182981, 1:1000), anti-APP (ABclonal, Cat# A17911, 1:100), anti-CD74 (ABclonal, Cat# A5667, 1:200), and anti-CD138/Syndecan-1 (ABclonal, Cat# A4174, 1:200). HRP-conjugated goat anti-rabbit IgG H&L (Abcam, Cat# ab6721, 1:1000) was used as the secondary antibody. After completion of all staining cycles, nuclei were counterstained with DAPI (Sigma, Cat# D9542), and sections were mounted with antifade mounting medium.

Single-plex staining was performed to optimize antibody dilution and signal separation, and sequential staining was conducted with microwave-mediated stripping between cycles to minimize antibody carryover. Images were acquired using an upright fluorescence microscope under consistent acquisition settings for each marker across comparable samples. For figure presentation, channels were pseudocolored as follows: CD31, green; APP, yellow; CD74, cyan; CD138, red; and nuclei, blue.

#### 2.9.2 Quantitative image analysis

Image quantification was performed in FIJI/ImageJ using batch-processed channel extraction and threshold-based segmentation. For each marker, raw images were converted to 16-bit grayscale, and calibrated thresholds were applied to generate binary masks. To ensure anatomical comparability between healthy and periodontitis sections, representative low-magnification images were displayed with a unified orientation of the sulcular/junctional epithelial region and adjacent connective tissue; when necessary, healthy control images were horizontally flipped for presentation only. This orientation adjustment was not applied to raw images used for quantitative analysis. Anatomical regions, including oral sulcular epithelium, junctional or pocket epithelium, oral epithelium, and adjacent connective tissue, were annotated on low-magnification images. Four quantitative metrics were predefined. For each animal, three non-overlapping high-power fields (400×) were imaged within vascularized periodontal connective tissue regions adjacent to the sulcular/junctional epithelial compartment and alveolar crest, and the mean of the three field values was used as the representative value for that animal, ensuring that each animal contributed a single independent data point (n = 9 per group) to the statistical analysis.

First, the APP enrichment ratio within endothelial regions was calculated as the mean APP intensity in CD31+ pixels divided by the mean APP intensity in CD31− pixels, thereby measuring endothelial attribution of APP. Second, the proportion of CD74+ cells among CD138+ plasma cells was calculated to quantify receptor expression within the plasma cell compartment. Third, the mean Euclidean distance from each CD138+ plasma cell centroid to the nearest CD31+ vascular pixel was measured to estimate plasma cell proximity to the vasculature. Fourth, the mean spatial distance from APP+CD31+ endothelial composite regions to the nearest CD74+CD138+ plasma cell composite regions was calculated to assess functional endothelial-plasma cell niche coupling.

Animal-level values (mean of three fields per animal; *n* = 9 per group) were compared between healthy and periodontitis groups using the Wilcoxon rank-sum test.

### 2.10 Statistical analysis and software

All computational analyses were conducted in R (version 4.3.1) unless otherwise specified. Major packages included limma (v3.56.2), Seurat (v4.3.0), Harmony (v0.1.1), scDblFinder (v1.14.0), clusterProfiler (v4.8.3), ReactomePA (v1.44.0), CellChat (v1.6.1), BisqueRNA (v1.0.5), igraph (v1.5.1), ggraph (v2.1.0), tidygraph (v1.2.3), pheatmap (v1.0.12), ggplot2 (v3.4.4), and ggpubr (v0.6.0). CIBERSORTx analyses were performed through the official web platform (https://cibersortx.stanford.edu), and image analysis was performed in FIJI/ImageJ (v1.54f). Unless otherwise stated, all tests were two-sided. Benjamini-Hochberg correction was applied for multiple testing where relevant, and adjusted *P* values below 0.05 were considered statistically significant.

## 3 Results

### 3.1 Transcriptomic landscape of peri-implantitis and periodontitis

**Figure 1:**
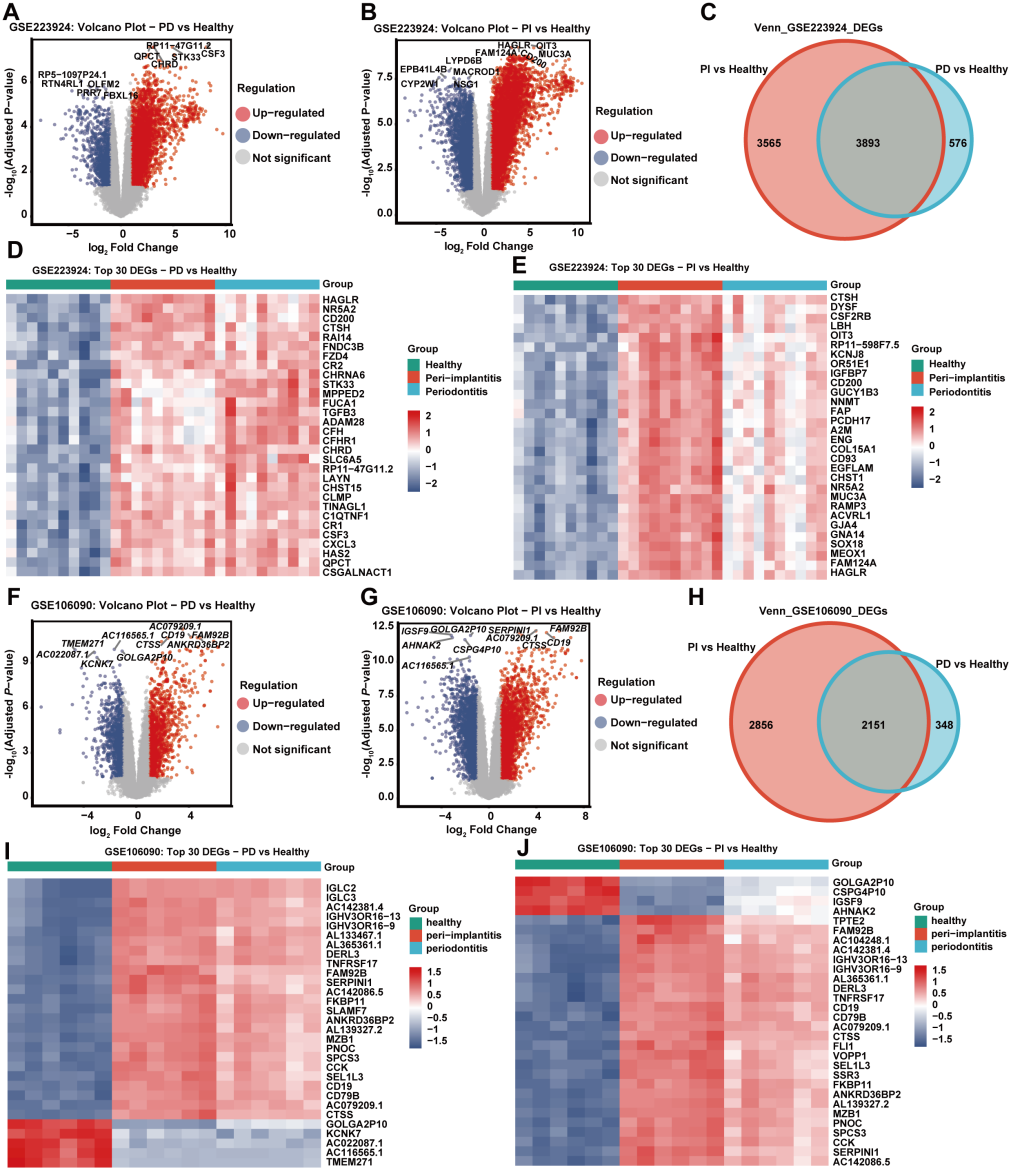
Transcriptomic profiling and cross - dataset identification of differentially expressed genes (DEGs) in peri - implantitis (PI) and periodontitis (PD). **(A–C)** Discovery transcriptomic analysis of the RNA-seq dataset GSE223924 (*n* = 10 per group). Volcano plots depicting DEGs in PD vs. healthy **(A)** and PI vs. healthy **(B)**. Each dot represents a gene; red, upregulated (logFC > 1, adj. *P* < 0.05); blue, downregulated (logFC < −1, adj. *P* < 0.05); gray, not significant. The top 5 most significantly upregulated and downregulated genes are labeled. Dashed lines indicate thresholds of |logFC| = 1 and adj. *P* = 0.05. **(C)** Venn diagram showing the overlap of DEGs between PI vs. healthy and PD vs. healthy comparisons in GSE223924, with 3,565 PI-specific, 576 PD-specific, and 3,893 shared DEGs. **(D, E)** Heatmaps displaying row-scaled expression of the top 30 DEGs (ranked by adjusted *P*-value) in PD vs. healthy **(D)** and PI vs. healthy **(E)** from GSE223924. Columns are ordered by group (healthy, peri-implantitis, periodontitis) without hierarchical column clustering. Color bars indicate group identity. **(F–H)** Validation transcriptomic analysis of the microarray dataset GSE106090 (n = 6 per group). Volcano plots of DEGs in PD vs. healthy **(F)** and PI vs. healthy **(G)**, following the same visualization conventions as **(A, B)**. **(H)** Venn diagram of DEGs between the two disease comparisons in GSE106090, revealing 2,856 PI-specific, 348 PD-specific, and 2,151 shared DEGs. **(I, J)** Heatmaps of the top 30 DEGs in PD vs. healthy **(I)** and PI vs. healthy **(J)** from GSE106090, presented as in **(D, E)**. Adj. *P*, Benjamini-Hochberg adjusted *P*-value; logFC, log_2_ fold change; PI, peri-implantitis; PD, periodontitis.

In the RNA-seq dataset GSE223924, limma-based analysis identified 7,458 DEGs in PI vs. H (4,530 upregulated, 2,928 downregulated) and 4,469 DEGs in PD vs. H (3,454 upregulated, 1,015 downregulated) (**Figures 1A, B**). Volcano plots highlighted the broader transcriptomic perturbation in PI compared with PD. Among the most significantly upregulated genes in PI were *MUC3A*, *FAM124A*, HAGLR, *OIT3*, and *CD200*, while *LYPD6B*, *EPB41L4B*, and *CYP2W1* were prominently downregulated (**Figure 1B**). Heatmap analysis of the top 30 DEGs revealed clear segregation of healthy, PI, and PD samples, with PI-associated DEGs predominantly showing upregulated angiogenic and immune-related genes (*ENG*, *ACVRL1*, *CSF2RB*, *CD93*) (**Figures 1D, E**). Within-dataset Venn analysis showed 3,893 DEGs shared between PI and PD, with 3,565 PI-specific and 576 PD-specific genes (**Figure 1C**).

In the independent microarray dataset GSE106090, PI vs. H yielded 5,007 DEGs (2,529 up, 2,478 down) and PD vs. H yielded 2,499 DEGs (1,388 up, 1,111 down) (**Figures 1F, G**). Consistently, PI demonstrated more extensive transcriptomic alteration than PD. B cell– and plasma cell–related genes (MZB1, *CD79B*, *TNFRSF17*, *DERL3*, *FKBP11*) were consistently enriched across both PI and PD relative to healthy controls (**Figures 1I, J**). Venn analysis in GSE106090 revealed 2,151 common DEGs between PI and PD, with 2,856 PI-specific and 348 PD-specific DEGs (**Figure 1H**).

### 3.2 Disease-specific divergence and shared inflammatory programs in periodontitis and peri-implantitis

Cross-dataset validation identified 820 PD-specific DEGs and 1,832 PI-specific DEGs after excluding the robust common DEGs shared by PD and PI (**Supplementary Figures 1A, E**). GO enrichment of PD-specific genes highlighted immune receptor signaling, leukocyte proliferation, and external side of plasma membrane as predominant terms (**Supplementary Figure 1B**). Reactome analysis revealed enrichment in immunoregulatory interactions between lymphoid and non-lymphoid cells, neutrophil degranulation, IL-10 signaling, and BCR activation (**Supplementary Figure 1C**). KEGG pathway analysis identified cytokine–cytokine receptor interaction, B cell receptor signaling, NF-κB signaling, and osteoclast differentiation (**Supplementary Figure 1D**).

PI-specific DEGs exhibited a notably broader enrichment profile. GO-BP terms additionally included leukocyte-mediated immunity and mononuclear cell proliferation (**Supplementary Figure 1F**). Reactome pathways uniquely enriched in PI included cell surface interactions at the vascular wall, FCGR activation, and parasitic infection pathways (**Supplementary Figure 1G**). KEGG analysis additionally highlighted NK cell-mediated cytotoxicity and cell adhesion molecule interaction pathways not observed among PD-specific enrichments (**Supplementary Figure 1H**).

**Figure 2:**
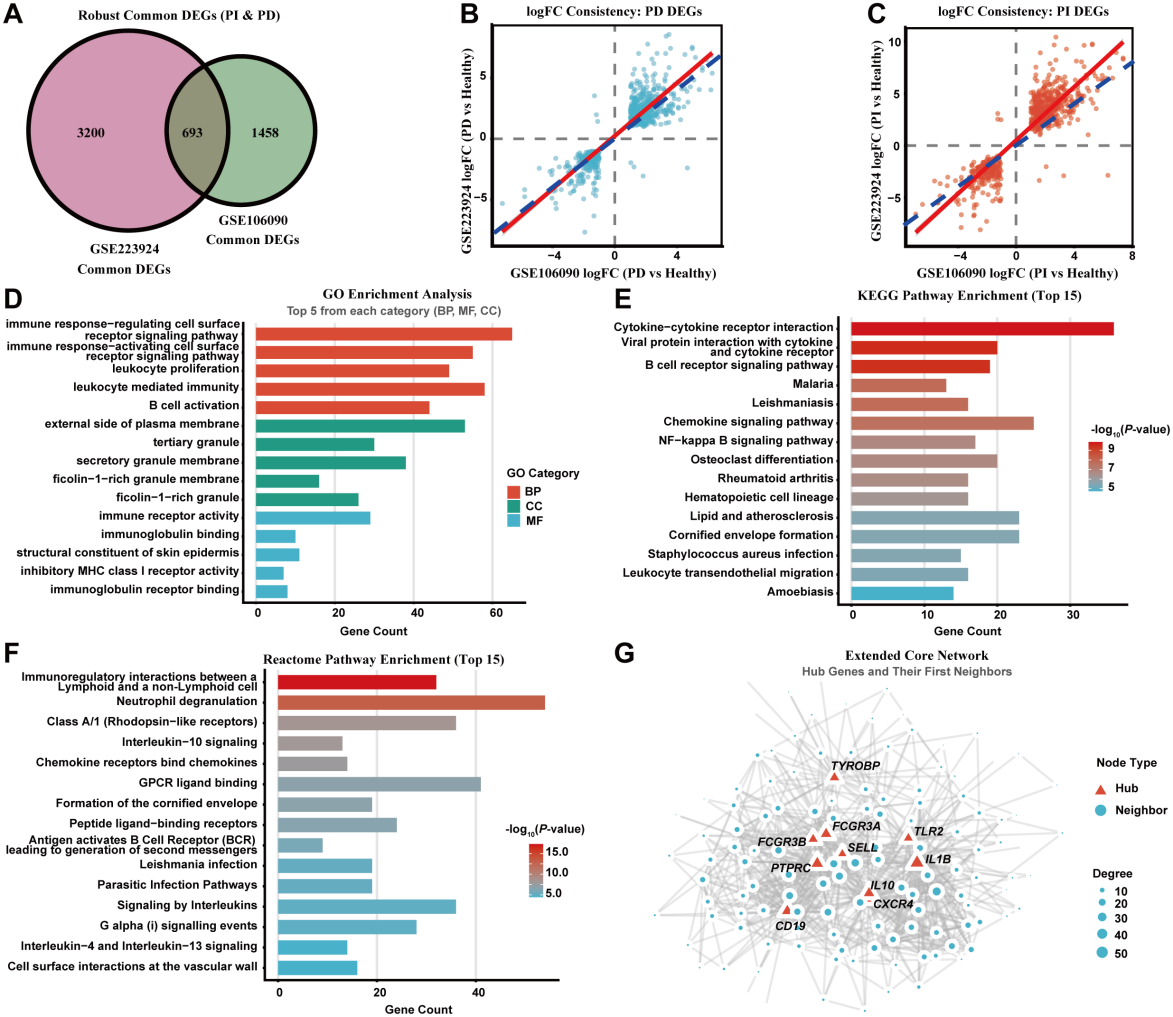
Functional enrichment and protein-protein interaction network analysis of robust cross-dataset common DEGs. **(A)** Venn diagram showing overlap of common DEGs identified in peri-implantitis (PI) and periodontitis (PD) between GSE223924 and GSE106090, yielding 693 robust common DEGs shared across both datasets. **(B)** Scatter plot showing logFC concordance of the 693 robust common DEGs in the PD-versus-healthy comparison between GSE106090 and GSE223924. **(C)** Scatter plot showing logFC concordance of the 693 robust common DEGs in the PI-versus-healthy comparison between GSE106090 and GSE223924. Each dot represents one gene; dashed lines indicate zero fold change, and diagonal trend lines indicate overall directional consistency. **(D)** GO enrichment analysis of the 693 robust common DEGs, showing the top five enriched terms from each ontology category: Biological Process (BP), Cellular Component (CC), and Molecular Function (MF). **(E)** KEGG pathway enrichment analysis showing the top 15 enriched pathways. **(F)** Reactome pathway enrichment analysis showing the top 15 enriched pathways. In **(E, F)**, bar color indicates enrichment significance as −log10(P-value). **(G)** Extended core protein-protein interaction (PPI) network of the top 10 hub genes and their first-degree neighbors. Hub genes are shown as red triangles, neighboring genes as blue circles, and node size is proportional to degree centrality. The network contains 134 nodes and 803 edges. DEG, differentially expressed gene; PI, peri-implantitis; PD, periodontitis; GO, Gene Ontology; KEGG, Kyoto Encyclopedia of Genes and Genomes; PPI, protein-protein interaction.

Because PD- and PI-specific DEGs revealed both overlapping immune themes and disease-context-dependent pathway divergence, we next focused on the robust common DEGs shared by PD and PI to define reproducible inflammatory programs across both disease states. Cross-dataset integration of GSE106090 and GSE223924 identified 693 robust common DEGs shared by peri-implantitis (PI) and periodontitis (PD) (**Figure 2A**). Of these, 686/693 (99.0%) showed concordant fold-change direction across datasets in both PD-versus-healthy and PI-versus-healthy comparisons, indicating high reproducibility (**Figures 2B, C**).

Enrichment analysis showed that these genes were predominantly involved in immune and inflammatory pathways. GO terms were mainly related to immune receptor signaling, leukocyte-mediated immunity, and B-cell activation (**Figure 2D**). KEGG analysis highlighted cytokine-cytokine receptor interaction, B-cell receptor signaling, chemokine signaling, NF-κB signaling, and osteoclast differentiation (**Figure 2E**), while Reactome analysis further emphasized immunoregulatory interactions, neutrophil degranulation, interleukin-10 signaling, and vascular wall-related cell surface interactions (**Figure 2F**).

The PPI network of the 693 robust common DEGs contained 277 nodes and 1,100 edges. The top 10 hub genes were IL1B, PTPRC, IL10, CD19, TLR2, FCGR3B, SELL, FCGR3A, CXCR4, and TYROBP, all upregulated in both PI and PD. The extended core network comprised 134 nodes and 803 edges, supporting a densely connected immune-centered interaction architecture (**Figure 2G**).

### 3.3 Single-cell transcriptomic atlas of gingival tissue reveals extensive cellular remodeling and cell type-specific localization of hub genes

**Figure 3:**
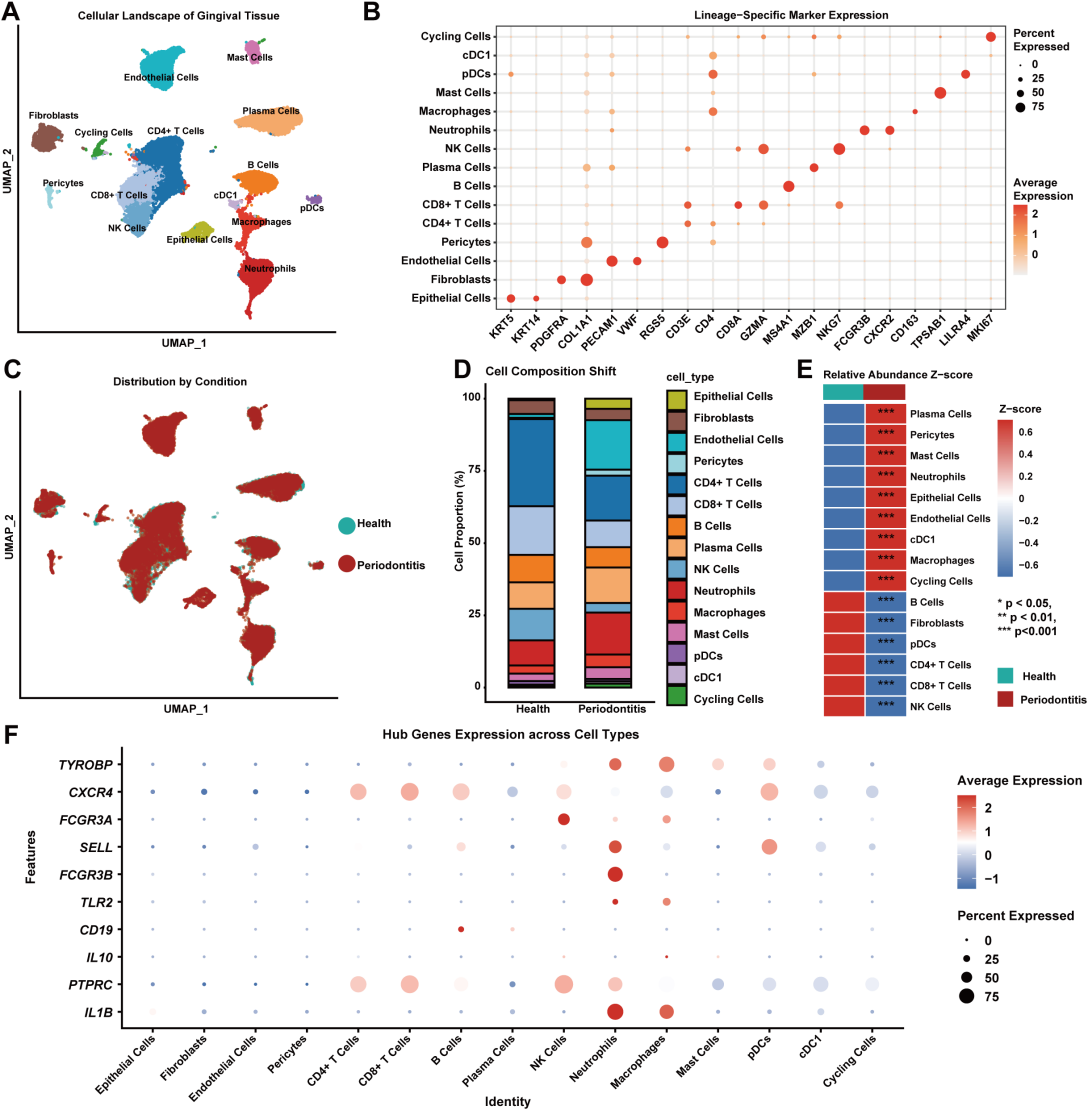
Single-cell transcriptomic landscape of healthy and periodontitis gingival tissue and cell type-specific expression of hub genes. **(A)** UMAP plot of 34,838 cells colored by annotated cell type, showing 15 major cellular populations identified after quality control, doublet removal, batch correction, and annotation. **(B)** Dot plot of canonical lineage markers across annotated cell populations, supporting cell type assignment. Dot size represents the percentage of cells expressing each marker, and dot color indicates average expression level. **(C)** UMAP plot colored by disease condition, showing the distribution of healthy and periodontitis-derived cells across the cellular landscape. **(D)** Stacked bar plot showing the relative proportions of annotated cell types in healthy and periodontitis tissues. **(E)** Heatmap showing row-scaled relative abundance (Z-score) of each cell population between healthy and periodontitis groups. Asterisks indicate the significance of compositional differences assessed by chi-square test (*P < 0.05; **P < 0.01; ***P < 0.001). **(F)** Dot plot showing the expression patterns of the top 10 hub genes across all annotated cell types. Dot size represents the percentage of expressing cells, and dot color indicates average expression level. UMAP, uniform manifold approximation and projection; pDCs, plasmacytoid dendritic cells; cDC1, conventional dendritic cells type 1.

Analysis of GSE171213 yielded 34,838 high-quality cells after quality control filtering and doublet removal, including 14,908 cells from healthy gingiva (42.8%) and 19,930 cells from periodontitis lesions (57.2%). Harmony-based integration effectively reduced sample-driven batch effects while preserving the underlying biological structure of the data. Resolution tuning supported the selection of 0.7 for downstream analysis, and the resulting clusters were annotated into 15 biologically interpretable cell populations on the basis of canonical lineage markers, including Epithelial Cells, Fibroblasts, Endothelial Cells, Pericytes, CD4+ T Cells, CD8+ T Cells, B Cells, Plasma Cells, NK Cells, Neutrophils, Macrophages, Mast Cells, pDCs, cDC1, and Cycling Cells (**Figures 3A, B**). Condition-level mapping further showed clear enrichment of periodontitis-derived cells in multiple immune-dominant regions of the UMAP space (**Figure 3C**).

Cell composition analysis revealed extensive remodeling of the gingival microenvironment in periodontitis. All 15 annotated cell populations differed significantly in relative abundance between healthy and periodontitis tissues (chi-square test, all *P* < 0.001; **Figures 3D, E**). Periodontitis was characterized by expansion of Plasma Cells, Pericytes, Mast Cells, Neutrophils, Epithelial Cells, Endothelial Cells, cDC1, Macrophages, and Cycling Cells, whereas Fibroblasts, CD4+ T Cells, CD8+ T Cells, B Cells, NK Cells, and pDCs were relatively depleted. Collectively, these findings indicate a marked shift from a stromal- and lymphocyte-enriched homeostatic state toward an inflammatory and tissue-remodeling cellular landscape in periodontitis.

To define the cellular context of the hub genes identified from the cross-dataset PPI network, we mapped their expression across all annotated cell types (**Figure 3F**). IL1B, TLR2, FCGR3B, and TYROBP were predominantly expressed in Neutrophils and Macrophages, consistent with activated innate immune compartments. PTPRC showed broad expression across leukocyte populations, whereas CD19 was largely restricted to B-lineage cells. CXCR4 was enriched mainly in lymphoid populations, and FCGR3A showed prominent expression in NK Cells. Overall, the hub genes were preferentially localized to immune cell populations expanded or activated in periodontitis, supporting the conclusion that the robust common inflammatory signature identified at the bulk transcriptomic level is primarily driven by disease-associated immune compartments at single-cell resolution.

### 3.4 Dual deconvolution analysis reveals shared and dataset-dependent cellular alterations in periodontitis and peri-implantitis

**Figure 4:**
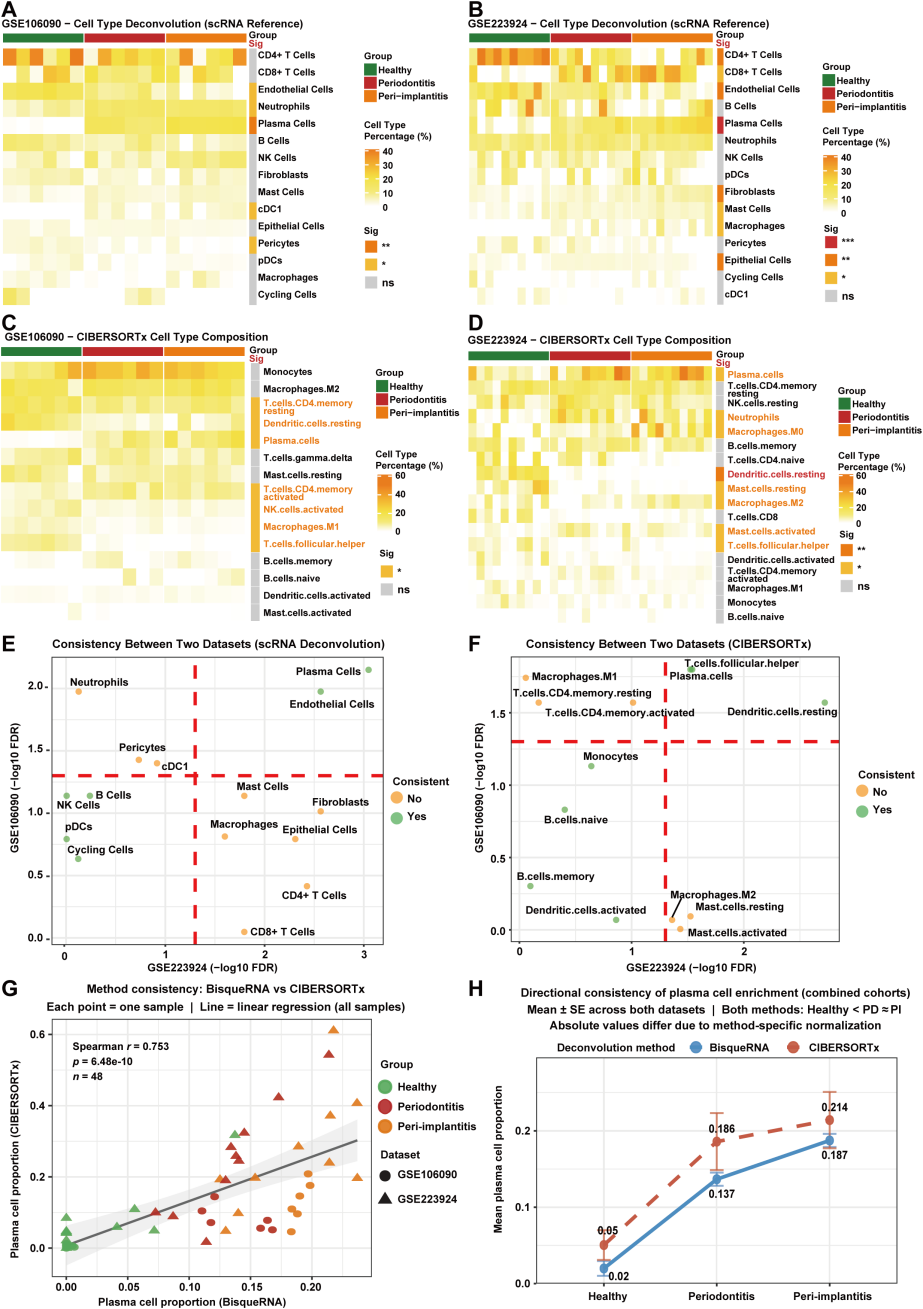
Dual deconvolution analysis reveals shared and dataset-dependent cellular alterations in periodontitis and peri-implantitis. **(A, B)** Heatmaps showing cell-type proportions estimated by BisqueRNA reference-based deconvolution using the GSE171213 single-cell atlas as reference, for GSE106090 **(A)** and GSE223924 **(B)**. Samples are ordered by group (Healthy, Periodontitis, and Peri-implantitis). Color intensity indicates estimated cell-type percentage. Row annotations indicate significance of overall group differences based on the Kruskal-Wallis test with BH correction. **(C, D)** Heatmaps showing immune cell fractions estimated by CIBERSORTx in GSE106090 **(C)** and GSE223924 **(D)**. Samples are displayed in the same group order as in **(A, B)**. Cell types with significant between-group differences are highlighted. **(E)** Cross-dataset comparison of BisqueRNA deconvolution results, showing −log10(FDR) values for each cell type in GSE223924 (x-axis) and GSE106090 (y-axis). Dashed red lines indicate the FDR = 0.05 threshold. Cell types located in the upper-right quadrant were significant in both datasets. **(F)** Cross-dataset comparison of CIBERSORTx results. Dashed red lines indicate the FDR = 0.05 threshold, and cell types in the upper-right quadrant were significant in both datasets. **(G)** Sample-level concordance of plasma cell proportions estimated by BisqueRNA and CIBERSORTx across matched samples from GSE106090 and GSE223924. Each point represents one sample; colors indicate clinical groups and shapes indicate datasets. The gray line represents linear regression across all samples with the shaded 95% confidence interval. Spearman correlation coefficient, p value, and sample size are indicated in the plot. **(H)** Directional consistency of plasma cell enrichment across the two deconvolution methods in the combined cohorts. Points and error bars indicate mean ± SE plasma cell proportions for each group and method. Although absolute proportions differ because of method-specific normalization, both BisqueRNA and CIBERSORTx show the same disease-associated trend, characterized by lower plasma cell proportions in healthy samples and higher proportions in PD and PI samples. PD, periodontitis; PI, peri-implantitis; SE, standard error; FDR, false discovery rate.

Using the GSE171213 single-cell atlas as a reference, we next deconvoluted the bulk transcriptomic datasets GSE106090 and GSE223924 to estimate cell-type composition across healthy, periodontitis (PD), and peri-implantitis (PI) samples. BisqueRNA reference-based deconvolution identified significant between-group differences in five cell types in GSE106090, including cDC1, Endothelial Cells, Neutrophils, Pericytes, and Plasma Cells (Kruskal-Wallis, FDR < 0.05; **Figure 4A**; **Supplementary Figure 2A**). In GSE223924, eight cell types differed significantly across groups, including CD4+ T Cells, CD8+ T Cells, Endothelial Cells, Epithelial Cells, Fibroblasts, Macrophages, Mast Cells, and Plasma Cells (**Figure 4B**; **Supplementary Figure 2B**). Cross-dataset comparison showed that Plasma Cells and Endothelial Cells were the only cell populations that remained significant in both cohorts, indicating partial but reproducible concordance across datasets (**Figure 4E**). Among these, Plasma Cells showed the most consistent disease-associated increase, particularly in PI samples.

To provide an orthogonal assessment of immune composition, we further applied CIBERSORTx. In GSE106090, seven immune populations showed significant group differences, including Plasma cells, resting and activated CD4 memory T cells, T follicular helper cells, activated NK cells, M1 macrophages, and resting Dendritic cells (**Figure 4C**; **Supplementary Figure 3A**). In GSE223924, eight populations were significantly altered, including Plasma cells, T follicular helper cells, M0 and M2 Macrophages, resting Dendritic cells, resting and activated Mast cells, and Neutrophils (**Figure 4D**; **Supplementary Figure 3B**). Cross-dataset consistency analysis identified Plasma cells, T follicular helper cells, and resting Dendritic cells as significant in both datasets (**Figure 4F**). Notably, Plasma cells were consistently enriched in disease samples, whereas T follicular helper cells and resting Dendritic cells tended to be reduced.

Taken together, the two deconvolution strategies revealed both convergence and heterogeneity in the inferred cellular landscapes of PD and PI. Plasma cell enrichment emerged as the most robust and reproducible feature across datasets and algorithms, whereas other inferred populations showed stronger dependence on cohort composition and deconvolution framework. To directly evaluate whether the two algorithms generated concordant plasma cell estimates, we further compared BisqueRNA- and CIBERSORTx-derived plasma cell proportions at the matched-sample level across both cohorts. The two methods showed a strong positive correlation for plasma cell proportions across all matched samples (Spearman *r* = 0.753, *p* = 6.48 × 10^-10^, *n* = 48; **Figure 4G**). Importantly, although the absolute values differed between methods because of method-specific normalization, both approaches preserved the same disease-associated directionality, with lower plasma cell proportions in healthy samples and higher proportions in PD and PI samples (Healthy < PD ≈ PI; **Figure 4H**). These findings support a shared humoral immune component in both PD and PI, superimposed on more context-dependent stromal and myeloid remodeling.

### 3.5 Plasma cell–centric communication network remodeling in periodontitis reveals enhanced endothelial coupling

**Figure 5:**
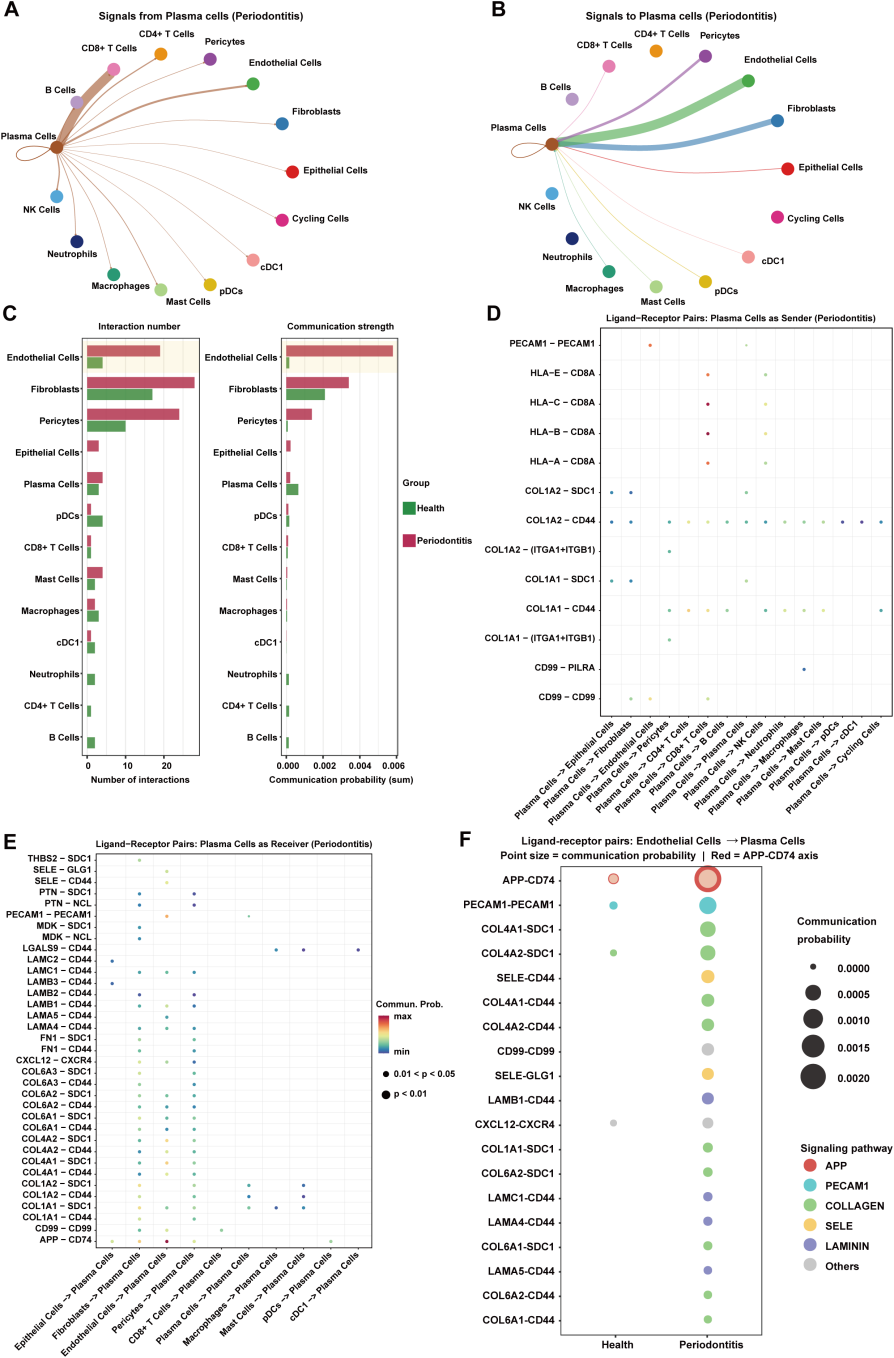
Plasma cell–centered intercellular communication network in periodontitis. **(A)** Circle plot showing outgoing communication from Plasma Cells to other cell populations in periodontitis. Edge thickness indicates the summed communication probability, and the self-loop denotes autocrine signaling. **(B)** Circle plot showing incoming communication to Plasma Cells from other cell populations in periodontitis. **(C)** Bar plots comparing the number of significant incoming ligand-receptor interactions and summed communication probability from each source cell population to Plasma Cells between healthy and periodontitis groups. Endothelial Cells showed a prominent increase in both interaction number and communication strength in periodontitis. **(D)** Bubble plot showing ligand-receptor interactions in which Plasma Cells act as senders in periodontitis. The y-axis lists ligand-receptor pairs, and the x-axis indicates target cell populations. Dot color represents communication probability, and dot size indicates statistical significance. **(E)** Bubble plot showing ligand-receptor interactions in which Plasma Cells act as receivers in periodontitis. The y-axis lists ligand-receptor pairs, and the x-axis indicates source cell populations. Dot color represents communication probability, and dot size indicates statistical significance. The endothelial-derived APP-CD74 interaction is the top-ranked incoming pair and is indicated to emphasize the specific endothelial-to-plasma cell axis prioritized for downstream validation. **(F)** Focused bubble plot of endothelial-to-Plasma Cell ligand-receptor interactions in healthy and periodontitis groups. Dot size represents communication probability, and dot color indicates the signaling pathway category. APP-CD74 is highlighted as the strongest endothelial-to-Plasma Cell interaction in periodontitis. LR, ligand-receptor.

Given the robust expansion of Plasma Cells across single-cell and deconvolution analyses, we next interrogated plasma cell-centered intercellular communication using CellChat. Under healthy conditions, Plasma Cells primarily acted as senders toward lymphocyte populations, with the strongest outgoing communication directed to CD8+ T Cells and, to a lesser extent, CD4+ T Cells (**Supplementary Figures 4A, D**). These outgoing signals were mainly mediated by MHC-I-related interactions, including HLA-A/B/C/E-CD8A, together with collagen-associated CD44 signaling. In periodontitis, this outgoing communication pattern was partially preserved but showed a notable shift toward Endothelial Cells (**Figures 5A, C**). In particular, PECAM1-PECAM1 homotypic adhesion emerged as a high-confidence interaction, accompanied by gain of CD99-CD99 signaling, indicating enhanced adhesive and vascular-interface communication from Plasma Cells in the diseased state.

A more striking remodeling was observed when Plasma Cells were analyzed as receivers. In health, incoming signals to Plasma Cells were derived predominantly from stromal compartments, especially Fibroblasts and Pericytes (**Supplementary Figures 4B, C**). These interactions were dominated by extracellular matrix- and chemokine-related pathways, including collagen-SDC1, CXCL12-CXCR4, and APP-CD74. In periodontitis, however, the incoming communication landscape shifted markedly toward Endothelial Cells, which became the dominant signaling source to Plasma Cells (**Figures 5B, D**). Quantitative comparison of incoming plasma cell-directed communication further confirmed this endothelial shift. Compared with health, endothelial-to-Plasma Cell interactions increased from 4 to 19 in periodontitis, accompanied by an approximately 7.7-fold increase in average communication probability and a marked increase in summed communication strength (**Figure 5C**). The strongest incoming interaction was APP-CD74 from Endothelial Cells, followed by PECAM1-PECAM1, collagen IV-SDC1/CD44, SELE-CD44/GLG1, and laminin-CD44 signaling. Focused analysis of endothelial-to-Plasma Cell ligand-receptor pairs showed that APP-CD74 was the top-ranked interaction in periodontitis and was markedly stronger than in health, supporting APP-CD74 as a candidate endothelial-plasma cell communication axis in periodontitis gingival tissue (**Figure 5F**). It should be emphasized that the prominence of APP-CD74 reflects the directed endothelial-to-plasma cell ligand-receptor pairing rather than a large change in either gene in isolation. CD74 is constitutively expressed by plasma cells and APP is broadly expressed by stromal and vascular populations, so at the bulk level neither gene alone necessarily shows the strongest disease-associated fold change. What changed in periodontitis was the cellular source of APP input to Plasma Cells, which shifted from a predominantly fibroblast- and pericyte-derived signal in health toward a dominant endothelial-derived signal in disease, together with expansion of the CD74-expressing plasma cell compartment; the combination of these two effects, rather than a single gene, drives the inferred increase in endothelial APP-to-plasma cell CD74 communication probability. This interpretation also clarifies why the supporting bulk transcriptomic evidence for this pair is incremental rather than dramatic and why the axis is most clearly resolved at the cell-resolved communication level. Notably, the CXCL12-CXCR4 axis remained detectable, originating from both Fibroblasts and Endothelial Cells.

Together, these findings indicate that plasma cell-centered communication is extensively rewired in periodontitis. Whereas healthy Plasma Cells are embedded mainly within a stromal-supportive microenvironment, periodontitis is characterized by pronounced endothelial coupling, with acquisition of vascular adhesion-, trafficking-, and matrix-associated signaling. These computationally inferred communication patterns suggest that Plasma Cells may occupy a more vascularly engaged inflammatory niche in periodontitis, with endothelial APP-CD74 signaling representing a prominent candidate axis for subsequent validation. To make this prioritization transparent, the plasma cell communication analysis was deliberately narrowed in stages: from the global plasma cell interactome across all populations (**Figures 5A, B**), to a source-resolved comparison that isolated Endothelial Cells as the dominant disease-associated input (**Figure 5C**), and finally to the ranked endothelial-to-plasma cell ligand-receptor pairs in which APP-CD74 emerged at the top (**Figures 5E, F**). The single APP-CD74 axis is therefore presented as the endpoint of an explicit, stepwise filtering process rather than a pre-selected interaction.

### 3.6 Independent periodontitis cohorts support the plasma cell-endothelial inflammatory program

To further evaluate the robustness of the plasma cell-endothelial inflammatory program, we first examined a predefined target gene panel in the original bulk datasets GSE106090 and GSE223924 and then validated these patterns in the independent periodontitis cohorts GSE10334 and GSE16134. The panel included hub genes, plasma cell markers, plasma cell-associated receptor genes, endothelial markers, and endothelial-associated ligand or matrix genes. Because of platform-specific gene coverage, 29 of 32 genes were detected in GSE106090, all 32 genes in GSE223924, and 30 genes in the integrated GSE10334/GSE16134 validation cohort. In the two original datasets, disease samples generally showed upregulation of hub genes, plasma cell markers, and endothelial-associated genes, with a more coherent disease-associated pattern in GSE223924 than in GSE106090 (**Supplementary Figures 5A, B**). Notably, CD74 was increased or significantly altered in disease samples, whereas CD44 was consistently reduced in both primary cohorts, suggesting divergent regulation of plasma cell-associated receptor components. Endothelial markers and matrix/adhesion-related genes, including PECAM1, CDH5/CD34, COL4A1, COL4A2, SELE, LAMA1, and LAMB1, were broadly altered, although APP showed dataset- and disease-context-dependent variation in the original three-group cohorts.

**Figure 6:**
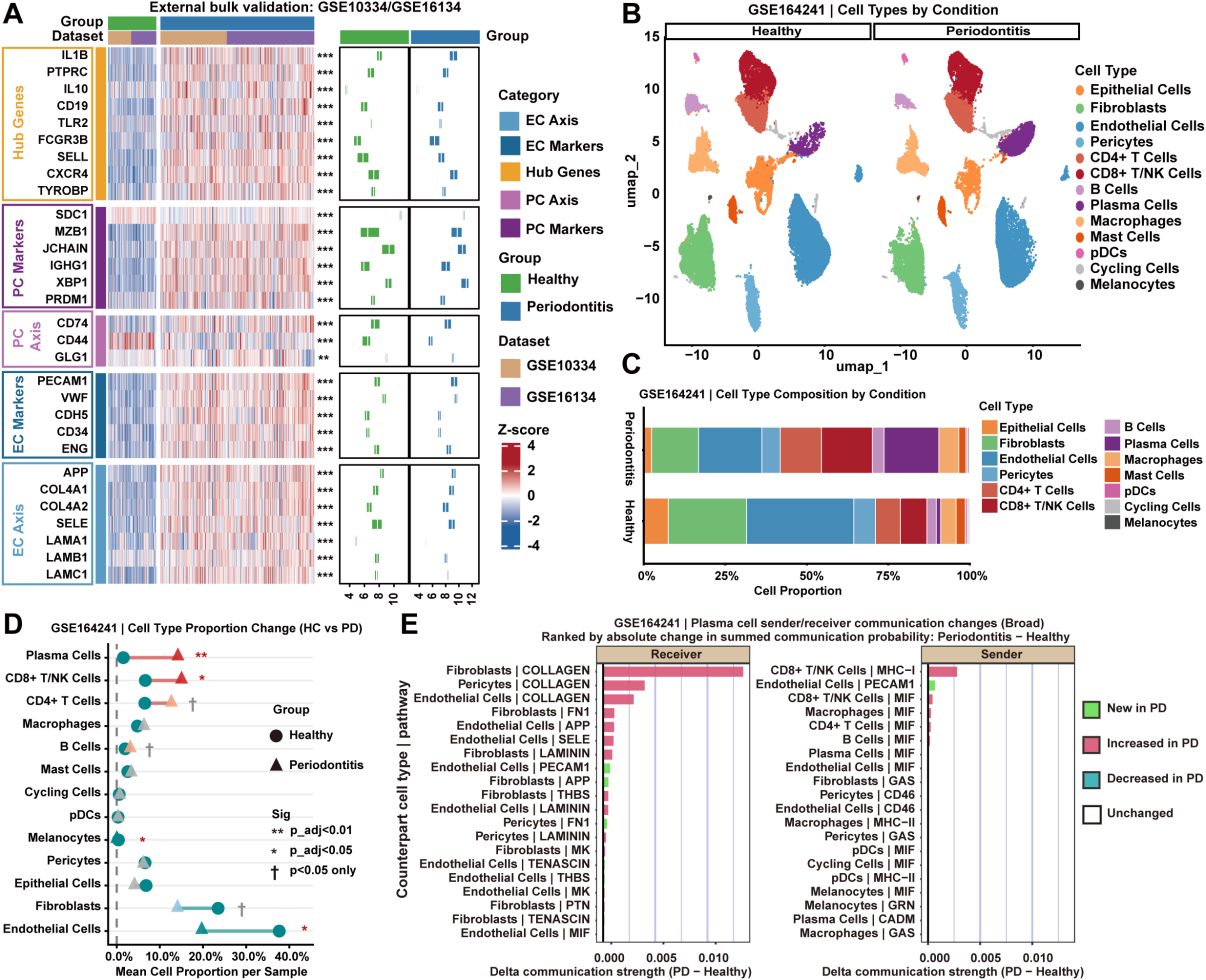
Independent periodontitis cohorts support plasma cell enrichment and plasma cell-endothelial communication remodeling. **(A)** Targeted gene-panel validation in the independent bulk cohorts GSE10334 and GSE16134. The integrated validation cohort included 557 gingival tissue samples, comprising 133 healthy and 424 periodontitis samples. The heatmap shows row-scaled expression of available hub genes, plasma cell markers, plasma cell-associated receptor genes, endothelial markers, and endothelial-associated ligand or matrix genes in healthy and periodontitis samples after dataset integration and batch correction. Right-side boxplots summarize group-level expression distributions. **(B)** UMAP visualization of broad cell-type annotations in the independent scRNA-seq dataset GSE164241 after exclusion of buccal mucosa samples, split by healthy and periodontitis conditions. After quality control and doublet removal, the final annotated dataset included 21 samples and 81,975 cells. **(C)** Stacked bar plot showing broad cell-type composition in healthy and periodontitis samples from GSE164241. **(D)** Sample-level comparison of broad cell-type proportions between healthy and periodontitis samples in GSE164241. Points indicate mean cell proportions per sample, and line segments indicate disease-associated changes. Statistical significance was assessed using Wilcoxon rank-sum tests with Benjamini-Hochberg correction. **(E)** Plasma cell-centered sender and receiver communication changes inferred by CellChat in GSE164241. Bars show changes in summed communication probability between periodontitis and healthy groups; positive values indicate increased communication in periodontitis. PD, periodontitis.

External validation using the independent periodontitis bulk cohorts GSE10334 and GSE16134 further supported this pattern. After integration and batch correction of GSE10334 and GSE16134, 557 gingival tissue samples were included, comprising 133 healthy and 424 periodontitis samples. In this large validation cohort, most hub genes, plasma cell-related genes, endothelial markers, and endothelial-associated ligand or matrix genes were significantly upregulated in periodontitis (**Figure 6A**). CD74 and GLG1 were increased, whereas CD44 was again significantly decreased, reproducing the receptor-level pattern observed in the original datasets. APP, which showed a more heterogeneous pattern in the smaller primary cohorts, was significantly upregulated in the large periodontitis validation cohort. Together, these targeted expression analyses support a reproducible vascular-immune transcriptional program in periodontitis, while indicating that individual axis genes such as APP may be more robustly validated in larger periodontitis-only cohorts than in smaller three-group discovery datasets.

We next validated cell-type alterations in GSE10334 and GSE16134 using two deconvolution strategies. CIBERSORTx showed a consistent increase in plasma cells in periodontitis in both datasets, and BisqueRNA independently identified Plasma Cells as one of the most significantly altered populations. Other immune and stromal populations showed more dataset-dependent variation, supporting plasma cell enrichment as the most reproducible cellular feature across deconvolution frameworks (**Supplementary Figure 5C**).

Finally, we analyzed the independent scRNA-seq dataset GSE164241 after excluding buccal mucosa samples. After quality control, doublet removal, and broad cell-type annotation, the final validation dataset contained 21 samples, including 13 healthy and 8 periodontitis samples, and 81,975 annotated cells. Broad cell-type annotation identified epithelial cells, fibroblasts, endothelial cells, pericytes, T/NK cells, B cells, plasma cells, macrophages, mast cells, pDCs, cycling cells, and melanocytes across healthy and periodontitis samples (**Figure 6B**). Cell-type composition analysis confirmed marked plasma cell expansion in periodontitis, increasing from 1.55% in healthy samples to 14.19% in periodontitis samples (adjusted *P* = 0.0098; **Figures 6C, D**).

CellChat analysis of GSE164241 further supported plasma cell-centered communication remodeling. In receiver mode, Plasma Cells received stronger signals in periodontitis from stromal and vascular compartments, particularly Fibroblasts, Pericytes, and Endothelial Cells. Increased incoming pathways included COLLAGEN, FN1, SELE, LAMININ, PECAM1, and APP-related interactions. In sender mode, Plasma Cells showed increased outgoing communication toward CD8+ T/NK Cells through MHC-I signaling, together with enhanced PECAM1- and MIF-related interactions involving endothelial and immune compartments (**Figure 6E**; **Supplementary Figure 5D**). Together, these independent bulk, deconvolution, and single-cell analyses corroborate a periodontitis-associated program characterized by plasma cell enrichment, endothelial activation, and strengthened plasma cell-centered vascular-immune communication.

### 3.7 Spatial transcriptomics supports periodontitis-associated APP–CD74 spatial co-enrichment and endothelial–plasma cell proximity

**Figure 7:**
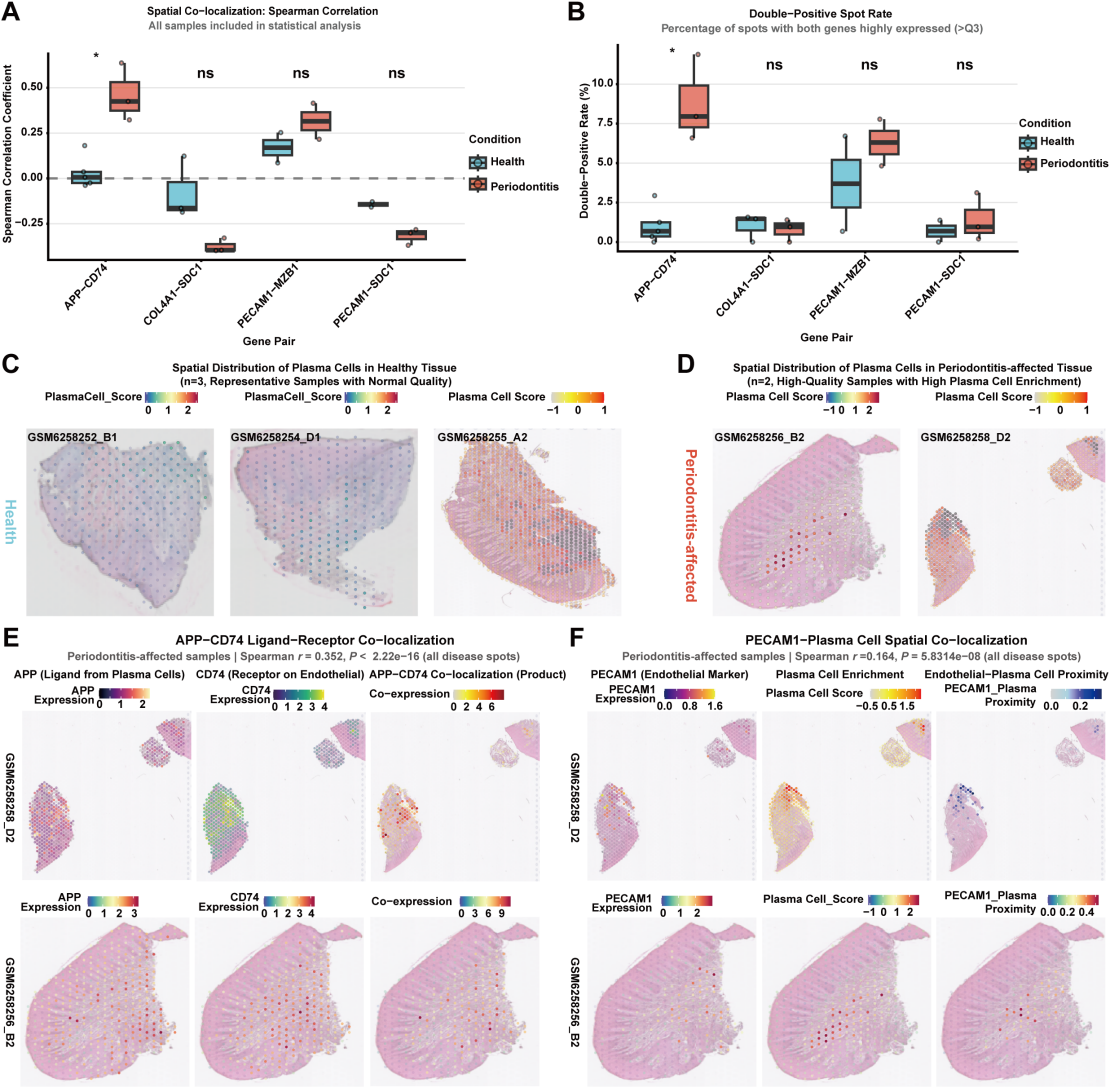
Spatial transcriptomic support for plasma cell-associated communication remodeling in periodontitis-affected oral mucosa. **(A)** Box plots showing sample-level Spearman correlation coefficients for four candidate gene pairs, including APP-CD74, COL4A1-SDC1, PECAM1-MZB1, and PECAM1-SDC1, in healthy and periodontitis-affected samples. Each point represents one Visium section. Statistical significance was assessed by Wilcoxon rank-sum test; *, *P* < 0.05; ns, not significant. **(B)** Box plots showing double-positive spot rates for the same gene pairs, defined as the percentage of spots in which both genes were highly expressed. Statistical testing was performed as in **(A)**. **(C)** Spatial feature plots showing plasma cell module scores in representative healthy samples, illustrating generally low and diffuse plasma cell enrichment. **(D)** Spatial feature plots showing plasma cell module scores in representative periodontitis-affected samples, illustrating focal accumulation of plasma cell-enriched regions. **(E)** Spatial maps of APP expression, CD74 expression, and their co-expression product in representative periodontitis-affected samples, supporting spot-level spatial co-enrichment of the APP-CD74 axis. Across all periodontitis-affected spots, APP and CD74 showed a significant positive correlation. **(F)** Spatial maps of PECAM1 expression, plasma cell module score, and the PECAM1-plasma cell proximity score in representative periodontitis-affected samples, showing close spatial association between endothelial-rich regions and plasma cell-enriched niches. SCT, SCTransform; Visium, 10x Genomics Visium spatial transcriptomics platform.

To spatially support the plasma cell-centered communication remodeling inferred from single-cell and CellChat analyses, we next analyzed GSE206621, a human oral mucosa spatial transcriptomic dataset including healthy and periodontitis-affected individuals. Sample-level co-localization analysis revealed a significant periodontitis-associated increase in APP-CD74 spatial coupling (**Figure 7A**). Specifically, the Spearman correlation coefficient increased from near zero in healthy tissue to a clearly positive level in periodontitis-affected oral mucosa (mean *r* = 0.032 ± 0.088 vs. 0.462 ± 0.160), corresponding to a 14.43-fold increase (Wilcoxon *P* = 0.036; Cohen’s d = 3.32). Consistently, the proportion of double-positive spots, defined as spots in which both genes were highly expressed, was markedly higher in periodontitis-affected samples than in health (**Figure 7B**). At pooled spot level across all periodontitis-affected sections, APP and CD74 also showed a significant positive correlation (Spearman *r* = 0.352, *P* < 2.22 × 10^-16^), further supporting spatial co-enrichment of this ligand-receptor pair in periodontitis-affected oral mucosa (**Figure 7E**).

We next examined the spatial distribution of Plasma Cell signals. In healthy tissue, plasma cell module scores remained uniformly low without evident focal enrichment, whereas periodontitis-affected tissue showed discrete regions with markedly elevated plasma cell signatures, indicating localized accumulation of plasma cell-rich niches (**Figures 7C, D**). To further evaluate the relationship between these plasma cell-enriched regions and the endothelial compartment, we quantified the spatial association between PECAM1 expression and plasma cell module enrichment. At pooled spot level across periodontitis-affected sections, PECAM1 expression showed a significant positive correlation with the plasma cell proximity score (Spearman *r* = 0.164, *P* = 5.8314 × 10^-8^), supporting close spatial apposition between endothelial-rich and plasma cell-enriched regions (**Figure 7F**).

By contrast, the other candidate pairs examined at sample level, including COL4A1-SDC1, PECAM1-MZB1, and PECAM1-SDC1, showed numerical shifts in periodontitis-affected tissue but did not reach statistical significance after multiple-testing correction (**Figures 7A, B**). Together, these spatial data provide *in situ* support for periodontitis-associated plasma cell niche remodeling, with APP-CD74 emerging as the most robust spatially supported candidate interaction and endothelial-plasma cell proximity representing an additional organizational feature of periodontitis-affected oral mucosa.

### 3.8 Multiplex immunofluorescence supports endothelial-plasma cell niche coupling involving APP-CD74 in a rat periodontitis model

**Figure 8:**
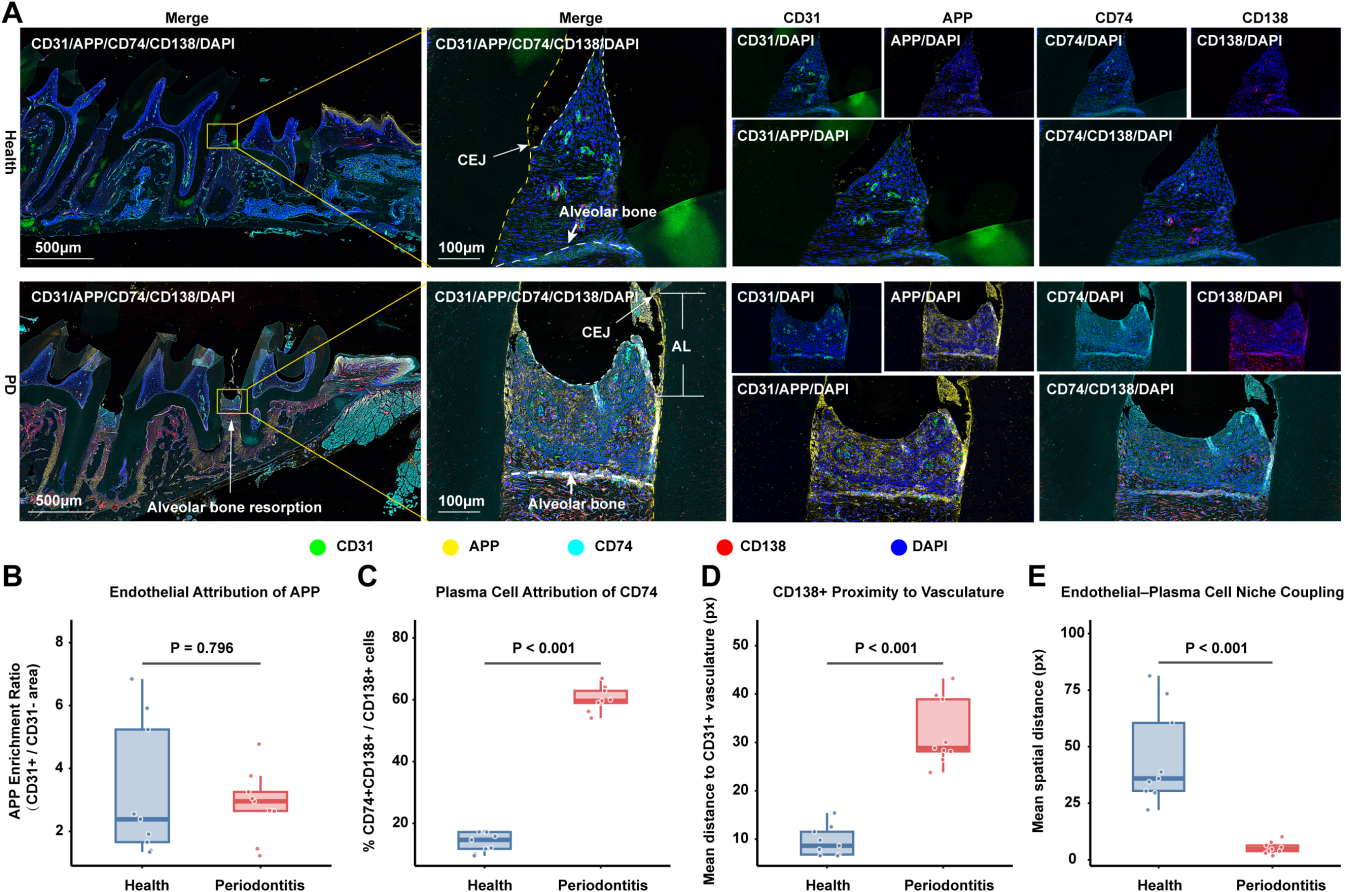
Multiplex immunofluorescence supports APP-CD74-associated endothelial-plasma cell spatial coupling in rat periodontitis tissue. **(A)** Representative four-color TSA multiplex immunofluorescence images of periodontal tissue sections from healthy control (Health, upper row) and periodontitis (PD, lower row) SD rats. For each group, the left panel shows a low-magnification merged image, and the boxed region is shown at higher magnification in the adjacent panel. The cementoenamel junction (CEJ) is indicated as an anatomical landmark. In PD tissue, alveolar bone resorption, alveolar bone, and attachment loss (AL) are annotated to confirm the establishment of experimental periodontitis. When necessary, healthy control images were horizontally flipped for standardized presentation only; all quantitative analyses were performed using the original unflipped images. Single-channel images for CD31, APP, CD74, and CD138, together with selected merged views, are shown on the right. CD31, green; APP, yellow; CD74, cyan; CD138, red; DAPI, blue. Scale bars: 500 μm and 100 μm. **(B)** APP enrichment ratio within CD31^+^ endothelial regions, calculated as APP intensity in CD31^+^ area relative to APP intensity in CD31^−^ area, showing stable endothelial attribution of APP between groups. **(C)** Percentage of CD74^+^ cells among CD138^+^ plasma cells, indicating CD74 receptor expression within the plasma cell compartment. **(D)** Mean Euclidean distance from CD138^+^ plasma cells to the nearest CD31^+^ vascular structure. **(E)** Mean spatial distance from APP^+^CD31^+^ endothelial composite regions to the nearest CD74^+^CD138^+^ plasma cell composite regions, reflecting endothelial–plasma cell niche coupling. In **(B–E)**, box plots show the median, interquartile range, and individual animal-level values (*n* = 9 rats per group; three non-overlapping fields per animal were averaged to yield one data point per animal). P values were determined by Wilcoxon rank-sum test.

To provide protein-level spatial support for the APP–CD74 endothelial–plasma cell communication pattern predicted by single-cell, spatial transcriptomic, and CellChat analyses, we performed four-color TSA multiplex immunofluorescence on periodontal sections from healthy control and periodontitis model SD rats, simultaneously labeling the endothelial marker CD31, the ligand APP, the receptor CD74, and the plasma cell marker CD138 (**Figure 8A**). To facilitate anatomical interpretation, representative low-magnification images were displayed in a standardized orientation across groups, and the cementoenamel junction (CEJ) was annotated as a key anatomical landmark in the magnified images. In PD tissue, interdental alveolar bone resorption was indicated in the overview image, while alveolar bone and attachment loss (AL) along the tooth surface were further annotated in the magnified image to clarify the periodontal lesion site used for high-magnification assessment.

Morphologically, healthy periodontal tissue showed relatively sparse CD31^+^ vascular structures, limited CD138^+^ plasma cell infiltration, and weak CD74 signal. In contrast, periodontitis tissue exhibited marked vascular remodeling with expanded CD31^+^ vascular networks and prominent plasma cell infiltration. At the morphological level, the PD overview image was selected from a representative specimen showing interdental alveolar bone resorption in the low-magnification panel, whereas the magnified panel further showed AL relative to the CEJ and the remaining alveolar bone at the base of the lesion (**Figure 8A**), confirming successful ligation-induced periodontitis. APP signal remained closely associated with endothelial-rich regions in both groups, whereas CD74 expression was more evident in CD138^+^ plasma cell-rich areas in periodontitis (**Figure 8A**). Notably, CD138^+^ plasma cell-rich areas in periodontitis were not confined to the tooth-associated epithelial or attachment-loss front; instead, they extended into vascularized connective tissue compartments, consistent with the formation of a chronic vascular–immune niche rather than a localized epithelial-front inflammatory infiltrate alone.

Quantitative image analysis further refined these observations. The APP enrichment ratio within CD31^+^ endothelial regions did not differ significantly between groups (*P* = 0.796; **Figure 8B**), indicating that APP maintained stable endothelial attribution rather than showing disease-specific redistribution. By contrast, the proportion of CD74^+^CD138^+^ cells among CD138^+^ plasma cells was markedly increased in periodontitis (*P* < 0.001; **Figure 8C**), supporting enhanced receptor availability for APP-derived signaling within the plasma cell compartment.

Spatial distance analysis revealed two distinct features. First, the mean distance from CD138^+^ plasma cells to the nearest CD31^+^ vessel was significantly greater in periodontitis than in health (*P* < 0.001; **Figure 8D**), indicating that plasma cell infiltration in diseased tissue became more extensive and spatially diffuse. This finding suggests that plasma cells were broadly redistributed throughout inflamed connective tissue rather than simply accumulating immediately adjacent to the sulcular or junctional epithelium. Second, and more importantly, the mean distance between APP^+^CD31^+^ endothelial composite regions and CD74^+^CD138^+^ plasma cell composite regions was dramatically reduced in periodontitis (*P* < 0.001; **Figure 8E**), demonstrating tighter spatial coupling of the functionally defined ligand-producing and receptor-expressing compartments.

Together, these findings provide protein-level *in situ* support for APP–CD74-associated endothelial–plasma cell spatial coupling in the rat periodontitis model. Although plasma cells become more broadly distributed within inflamed tissue, the APP-expressing endothelial niche and the CD74-expressing plasma cell niche show significantly enhanced spatial apposition, supporting disease-associated reinforcement of this communication axis. Thus, the mIF data indicate that periodontitis-associated plasma cell niches are preferentially organized within vascularized connective tissue regions and are spatially coupled to APP^+^ endothelial structures, complementing the spatial transcriptomic evidence of endothelial-plasma cell proximity.

## 4 Discussion

Periodontitis and peri-implantitis are increasingly recognized not merely as two distinct clinical entities but as related inflammatory phenotypes sharing a core host response yet differing in magnitude and tissue context. Understanding the immune architecture that these conditions share—and the cellular niches that sustain destructive inflammation—is central to developing rational host-modulation strategies, a priority articulated in the 2023 EFP S3-level clinical practice guideline for peri-implant diseases (5).

This study integrated bulk transcriptomics, single-cell transcriptomics, dual deconvolution, cell–cell communication inference, spatial transcriptomics, and multiplex immunofluorescence to dissect the shared and distinct immune landscapes of periodontitis and peri-implantitis. Three major conclusions emerge. First, PI exhibited broader transcriptomic perturbation than PD, yet both diseases converged on a highly reproducible common inflammatory core. Second, plasma cell enrichment was the most stable and cross-validated cellular feature across datasets and analytical frameworks. Third, within periodontitis, orthogonal single-cell, spatial, and protein-level evidence supported a disease-associated endothelial–plasma cell niche remodeling program, with APP–CD74 emerging as the strongest candidate communication axis.

A consistent observation across both bulk transcriptomic cohorts was that PI displayed a larger number of DEGs than PD relative to healthy controls. This finding is compatible with prior histopathologic and clinicopathologic observations indicating that peri-implantitis lesions are often larger and more inflammatory than periodontitis lesions (14, 27, 28). It is also concordant with recent comparative transcriptomic studies showing broader activation of immune, vascular, and stromal programs in PI than in PD (17, 18). In our data, however, this broader perturbation did not indicate a qualitatively separate disease class. Instead, PI and PD shared 693 robust common DEGs with near-complete directional concordance across platforms, indicating that the two conditions rest on a strongly overlapping inflammatory transcriptional backbone.

The disease-specific enrichment patterns nevertheless suggest important biological differences. PD-specific signals were relatively concentrated in immune receptor signaling, B-cell activation, cytokine pathways, and osteoclast-associated inflammation, whereas PI-specific signatures extended further into vascular interaction, cytotoxic, and Fc receptor-related pathways. This pattern is biologically plausible in the peri-implant context, where implant-associated material factors, altered tissue architecture, and foreign body-associated immune modulation may amplify innate inflammatory responses beyond those seen in tooth-associated lesions (15, 16). Even so, the present results support a model of quantitative divergence on a shared inflammatory framework, rather than two wholly distinct immunopathologic processes.

The robust common DEG network was strongly enriched for immune receptor signaling, leukocyte-mediated immunity, cytokine–cytokine receptor interaction, chemokine signaling, and osteoclast differentiation, all of which are central themes in periodontal tissue destruction (9, 29, 30). Within this network, IL1B emerged as the highest-degree hub, consistent with its established role as a master pro-inflammatory mediator in periodontal bone loss and tissue degradation (11, 29). TLR2, FCGR3A, FCGR3B, CXCR4, TYROBP, and PTPRC further emphasized convergence on innate immune activation, leukocyte trafficking, and antibody-linked effector biology.

Single-cell mapping clarified the cellular context of this shared network. IL1B, TLR2, FCGR3B, and TYROBP were concentrated primarily in neutrophils and macrophages, whereas CD19 localized to the B-lineage compartment and CXCR4 was enriched mainly in lymphoid cells. These results argue that the shared molecular foundation of PD and PI is not merely a diffuse tissue-level inflammatory signature, but is anchored in specific immune cell populations with complementary functions: myeloid cells contributing cytokine amplification and innate effector signaling, and B-lineage cells contributing humoral inflammatory activity. This interpretation is also consistent with recent tissue-based analyses showing strong B-cell/plasma-cell representation and heightened inflammatory mediator profiles in destructive periodontal and peri-implant lesions (6, 14).

Among all inferred cell populations, plasma cells emerged as the most reproducible disease-associated feature. They were expanded in the periodontitis scRNA-seq atlas, recurrently elevated in BisqueRNA deconvolution, and independently supported by CIBERSORTx across both bulk cohorts. This convergence is notable because other inferred populations showed substantially stronger dependence on cohort composition and deconvolution framework. In other words, while endothelial, stromal, mast-cell, macrophage, or T-cell estimates varied across methods, plasma-cell enrichment remained robust. This aligns well with the longstanding histopathologic recognition that advanced periodontal lesions are plasma-cell-rich and with newer evidence indicating that peri-implantitis lesions are likewise enriched in B-cell/plasma-cell-associated immune programs (6, 14, 31).

At the same time, our study also highlights the importance of interpreting compositional analyses across biological scales. Single-cell proportions, bulk deconvolution estimates, and spatial abundance patterns do not measure exactly the same biological quantity and are sensitive to differences in sampling depth, denominator effects, lesion topography, and reference composition. This likely explains why endothelial-related signals appeared recurrently, but not always with the same inferred direction across scRNA-seq, BisqueRNA, and CIBERSORTx. Rather than indicating contradiction, such discrepancies more likely reflect vascular remodeling occurring at multiple levels, including changes in endothelial state, relative abundance, and regional organization. Against this background of method-dependent variability, the stability of the plasma-cell signal becomes even more compelling. The two deconvolution frameworks were not, however, treated as equivalent. BisqueRNA was used as the primary basis for our cellular conclusions because it is reference-based and was anchored to the matched gingival single-cell atlas generated in this study, allowing it to resolve the full set of annotated stromal, vascular, and immune populations, including non-hematopoietic compartments such as endothelial cells, fibroblasts, and pericytes that are central to our hypothesis. CIBERSORTx, which relies on the blood-derived LM22 immune signature and does not model non-immune lineages, was used as an orthogonal, immune-focused confirmation rather than as a co-equal estimate of tissue composition (32). The two methods also differ in normalization, which is why their absolute proportions are not directly comparable. Reassuringly, the conclusion that does not depend on this distinction—the disease-associated enrichment of plasma cells—was reproduced by both methods and across all cohorts, whereas inferences about non-plasma-cell populations rest preferentially on the reference-based BisqueRNA estimates.

The recurrent reduction of T follicular helper-like signals in CIBERSORTx, together with strong plasma-cell enrichment, is also intriguing. Although this should be interpreted cautiously given the limitations of immune deconvolution in non-hematopoietic tissues, it raises the possibility that plasma-cell accumulation in destructive lesions may not simply reflect classical germinal-center help, but could also involve extra-follicular or tissue-adapted B-cell differentiation programs. This possibility warrants direct investigation in future scRNA-seq studies that include peri-implantitis.

The most conceptually novel finding of this study is the identification, in periodontitis, of an endothelial–plasma cell communication program centered on APP–CD74. In CellChat analysis, endothelial cells became the dominant incoming signal source for plasma cells in disease, and APP–CD74 was the strongest individual candidate interaction in the plasma-cell receiver network. This result is especially noteworthy because healthy plasma cells were predicted to receive signals mainly from stromal populations, particularly fibroblasts and pericytes, whereas diseased plasma cells shifted toward a vascularly engaged niche.

The biological plausibility of this axis is supported by prior work outside the periodontal field. APP–CD74 signaling has recently been implicated in endothelial–macrophage communication during kidney injury and fibrosis, and APP–CD74 has also been identified as a functionally relevant immune-regulatory interaction in glioblastoma-associated macrophage biology (33, 34). On the receptor side, CD74 is not merely an antigen-presentation chaperone; cell-surface CD74 can initiate signaling programs linked to NF-κB activation, proliferation, survival, and CD44-dependent downstream signaling in B-lineage cells (35–39). On the ligand side, APP is expressed by endothelial cells and has been linked to endothelial activation, Src-dependent signaling, VCAM-1 induction, and monocyte adhesion (40, 41). Taken together, these studies do not prove that APP–CD74 is functionally active in periodontal plasma cells, but they do provide a coherent mechanistic framework for why an endothelial APP/plasma-cell CD74 interaction would be biologically meaningful in chronic inflammatory tissue.

Crucially, the present data support this axis as a candidate disease-associated communication program in PD, not as a definitively proven biochemical signaling mechanism in both PD and PI. Our direct communication inference, spatial validation, and rat tissue-level protein validation were all strongest in periodontitis. The relevance of the same axis to PI is plausible in light of the shared plasma-cell-rich inflammatory architecture of PI, but remains inferential in the absence of direct single-cell and spatial peri-implantitis validation. Several considerations further temper this extrapolation, because the two diseases are mechanistically related but not identical. Peri-implant lesions are additionally shaped by implant-derived factors such as titanium particle release and a foreign body-type tissue response, which have been implicated in macrophage activation, cytokine amplification, and vascular remodeling (15, 16). Given that APP is an endothelial activation-associated ligand and that CD74 signaling is responsive to the inflammatory milieu, the endothelial–plasma cell APP–CD74 axis could plausibly be preserved or even further reinforced in peri-implantitis; alternatively, titanium-driven myeloid and foreign body responses might redirect the dominant plasma cell inputs toward macrophage- or matrix-derived signals and thereby alter the relative prominence of this axis. These competing possibilities cannot be resolved from a periodontitis-only dataset. Moreover, our animal validation used a ligature-induced rat periodontitis model, which faithfully reproduces plaque retention-driven inflammation and alveolar bone loss, as confirmed by the visible alveolar bone resorption and deepened pocket in the representative PD specimen shown in **Figure 8A** (42). It is important to note that in ligation-induced models, the periodontal pocket develops through mechanical disruption and plaque retention at the ligature site rather than through the gradual apical migration of junctional epithelium that characterizes naturally progressing periodontitis; consequently, the pocket morphology and keratinized gingival contour may differ from those in spontaneous clinical disease (42, 43). Nevertheless, the cardinal disease features of our model, including inflammatory cell infiltration, vascular remodeling, and alveolar bone loss, are consistent with established ligature-induced periodontitis benchmarks (42, 44). The model, by design, cannot reproduce every aspect of human disease and does not incorporate the implant–tissue interface or titanium particle exposure that characterize peri-implantitis (45, 46). Determining whether APP–CD74 is enhanced, preserved, or remodeled in peri-implantitis will therefore require dedicated peri-implant single-cell, spatial, and, ideally, implant-bearing experimental models.

APP–CD74 did not emerge in isolation. CellChat further suggested a broader endothelial–plasma cell niche program in periodontitis involving selectin-associated, extracellular matrix-associated, and endothelial adhesion-related interactions. Endothelial inputs to plasma cells included SELE–CD44/GLG1, collagen IV–SDC1/CD44, laminin–CD44, and persistent CXCL12–CXCR4 signaling. These interactions are conceptually consistent with a model in which activated endothelium participates in plasma-cell recruitment, positioning, and retention. E-selectin has recognized relevance in periodontal vascular inflammation and can mediate endothelial interactions with periodontal pathogens and leukocyte trafficking programs (47, 48). PECAM1 and CD99, meanwhile, are established endothelial adhesion molecules involved in leukocyte transendothelial migration and vascular inflammatory biology (49, 50).

Our orthogonal validation data refine this model in an important way. In spatial transcriptomics, APP–CD74 showed the strongest disease-associated co-localization among the tested candidate pairs, while plasma-cell module enrichment formed discrete focal niches that were spatially associated with endothelial signal. In multiplex immunofluorescence, APP remained endothelium-associated in both healthy and diseased tissue, but CD74 expression within the CD138^+^ plasma-cell compartment increased markedly in periodontitis. Moreover, the distance between APP+CD31+ endothelial domains and CD74^+^CD138^+^ plasma-cell domains was dramatically reduced in periodontitis, even though the average distance of all CD138^+^ plasma cells to blood vessels increased. This distinction is important. It suggests that disease reinforcement of the APP–CD74 axis is driven less by disease-specific reassignment of APP to endothelium and more by plasma-cell receptor acquisition plus spatial niche reorganization, resulting in tighter coupling between functionally defined endothelial and plasma-cell microdomains.

This interpretation also helps reconcile the seeming paradox between broader plasma-cell infiltration and stronger endothelial–plasma cell coupling. In inflamed tissue, plasma cells appear to become more diffusely distributed overall, as reflected by their increased mean distance to the nearest CD31^+^ vascular structure, but a subset of plasma-cell-rich regions forms especially close spatial apposition to endothelial APP-rich niches. Thus, the key disease feature is not simple perivascular crowding of all plasma cells, but the emergence of specialized endothelial–plasma cell neighborhoods consistent with sustained inflammatory communication. This spatial organization is also consistent with classical periodontal histopathology and argues against a sampling or orientation artifact. Although neutrophils and the acute infiltrate dominate immediately beneath the sulcular and pocket epithelium, the chronic plasma-cell-rich infiltrate of established lesions is characteristically located deeper within the vascularized gingival connective tissue rather than at the epithelial front. The observation that CD138^+^ plasma cells in our sections were organized within vascularized connective tissue, rather than concentrated at the sulcular margin, therefore reflects the expected anatomy of a chronic plasma-cell lesion and reinforces, rather than undermines, the relevance of an endothelial–plasma cell niche.

These findings have potential translational implications, but they should be framed cautiously. The APP–CD74 axis is best viewed at present as a testable therapeutic hypothesis rather than an immediately actionable periodontal drug target. CD74 has been explored pharmacologically in other contexts—the anti-CD74 monoclonal antibody milatuzumab has reached Phase I evaluation in B-cell malignancies, though not in inflammatory disease (51) —and MIF/CD74 pathway inhibition continues to attract interest in chronic inflammatory and autoimmune conditions (52). In a periodontal context, the practical value of our findings lies less in immediate therapeutic translation than in the identification of a mechanistically coherent vascular–immune interface that may enrich future host-modulation strategies alongside standard mechanical debridement and emerging resolution-based approaches (45, 53). Direct functional studies are still required to determine whether perturbing APP–CD74 in periodontal tissues reduces plasma-cell persistence, inflammatory mediator production, or bone destruction. Such studies should test the predicted interaction directly, for example by stimulating plasma cells or plasma cell-like lines with recombinant APP and measuring downstream NF-κB activation, by loss-of-function approaches using CD74-neutralizing antibodies or CD74 knockdown, and by endothelial–plasma cell co-culture systems, as well as by confirmation in human gingival tissue, for example through qRT-PCR or *in situ* analysis of APP and CD74 across healthy, periodontitis, and peri-implantitis samples. CellChat infers communication from ligand-receptor co-expression and prior interaction knowledge and does not measure ligand binding or downstream signaling; accordingly, and in the absence of such functional and human experiments, the present results should be interpreted as correlative evidence consistent with, but not proof of, a functionally active APP-CD74 signaling axis.

More broadly, the present network view suggests that targeting a single ligand–receptor pair may be insufficient if endothelial–plasma cell coupling is sustained by a multicomponent niche program involving APP–CD74, selectins, matrix ligands, and endothelial adhesion molecules. Combination strategies that modulate innate inflammatory amplification together with stromal/vascular retention cues may therefore be more effective. In this context, host-modulation approaches aimed at restoring resolution rather than simply suppressing inflammation remain attractive, including pro-resolving mediator-based strategies that have shown promise in periodontal research (45, 53).

Several limitations should be acknowledged. First, the core human mechanistic inference relied on public datasets, and direct biochemical confirmation of APP–CD74 signaling in periodontal plasma cells was not performed. Second, the scRNA-seq reference dataset included healthy and periodontitis samples only, so cell-state mapping and CellChat inference could not be performed directly for peri-implantitis. Third, the spatial transcriptomic cohort was small, limiting statistical power for candidate pairs beyond APP–CD74. Fourth, CellChat predicts communication from transcriptomic co-occurrence and prior ligand–receptor knowledge, but does not directly measure ligand binding or downstream signaling. Fifth, the tissue-level validation was performed in a ligature-induced rat periodontitis model, which reproduces key features of disease (plaque accumulation, neutrophil influx, and bone loss) but compresses disease chronology and may not fully recapitulate the microbial dysbiosis or longitudinal remodeling of human PD, and does not replicate the implant–tissue interface relevant to PI. Finally, the study was cross-sectional, preventing inference about the temporal order by which endothelial remodeling and plasma-cell accumulation become coupled during lesion progression.

Despite these limitations, the convergence of evidence across bulk transcriptomics, single-cell mapping, dual deconvolution, CellChat, spatial transcriptomics, and multiplex immunofluorescence strongly supports the existence of a plasma-cell-centered inflammatory niche in destructive periodontal disease. Within that niche, endothelial–plasma cell coupling appears to be a previously underappreciated organizational principle, and APP–CD74 emerges as its most compelling candidate molecular axis in periodontitis. From a clinical perspective, these observations support incorporating vascular–plasma cell niche assessment into future mechanistic investigations of treatment-resistant lesions, and motivate prospective evaluation of whether anti-niche strategies can complement existing non-surgical and surgical approaches recommended in current EFP guidelines (5).

## 5 Conclusion

This integrative multi-omics study provides a comprehensive molecular and cellular characterization of the shared and divergent immune landscapes of periodontitis and peri-implantitis. By combining cross-platform bulk transcriptomics, single-cell RNA sequencing, dual deconvolution analysis, cell–cell communication inference, spatial transcriptomics, and tissue-level multiplex immunofluorescence, we identified plasma cell enrichment as the most reproducible immune infiltration feature across datasets and analytical frameworks. The central novel finding of this study is the identification, in periodontitis, of a disease-associated endothelial–plasma cell communication program centered on APP–CD74, supported by convergent single-cell, spatial, and protein-level evidence. Together with selectin-associated, matrix-associated, and PECAM1-related interactions, these results support a model of endothelial–plasma cell niche remodeling that may contribute to the maintenance of plasma cell-rich inflammatory lesions in destructive periodontal disease. These findings nominate gingival vascular–immune crosstalk as a testable mechanistic and translational target in chronic periodontal inflammation. The relevance of this endothelial–plasma cell signaling program to peri-implantitis is suggested by the shared inflammatory architecture of the two conditions but will require direct single-cell and spatial validation in peri-implant tissues. Ultimately, these insights position endothelial–plasma cell niche biology as a candidate axis for future host-modulatory strategies that may complement the established mechanical and surgical approaches endorsed by current EFP clinical practice guidelines.

## Data Availability

The raw data supporting the conclusions of this article will be made available by the authors, without undue reservation. All datasets analyzed in this study are publicly available in the Gene Expression Omnibus (GEO) under accession numbers GSE223924, GSE106090, GSE171213, GSE206621, GSE10334, GSE16134, and GSE164241.

## References

[1] Bernabe E Marcenes W Abdulkader RS Abreu LG Afzal S Alhalaiqa FN . Trends in the global, regional, and national burden of oral conditions from 1990 to 2021: a systematic analysis for the Global Burden of Disease Study 2021. Lancet. (2025) 405:897–910. doi: 10.1016/s0140-6736(24)02811-3 40024264

[2] Chen MX Zhong YJ Dong QQ Wong HM Wen YF . Global, regional, and national burden of severe periodontitis, 1990-2019: an analysis of the Global Burden of Disease Study 2019. J Clin Periodontol. (2021) 48:1165–88. doi: 10.1111/jcpe.13506 34101223

[3] Diaz P Gonzalo E Villagra LJG Miegimolle B Suarez MJ . What is the prevalence of peri-implantitis? A systematic review and meta-analysis. BMC Oral Health. (2022) 22:449. doi: 10.1186/s12903-022-02493-8 36261829 PMC9583568

[4] Galarraga-Vinueza ME Pagni S Finkelman M Schoenbaum T Chambrone L . Prevalence, incidence, systemic, behavioral, and patient-related risk factors and indicators for peri-implant diseases: an AO/AAP systematic review and meta-analysis. J Periodontol. (2025) 96:587–633. doi: 10.1002/jper.24-0154 40489307 PMC12273760

[5] Herrera D Berglundh T Schwarz F Chapple I Jepsen S Sculean A . Prevention and treatment of peri-implant diseases-the EFP S3 level clinical practice guideline. J Clin Periodontol. (2023) 50 Suppl 26:4–76. doi: 10.1111/jcpe.13823 37271498

[6] Malmqvist S Clark R Johannsen G Johannsen A Boström EA Lira-Junior R . Immune cell composition and inflammatory profile of human peri-implantitis and periodontitis lesions. Clin Exp Immunol. (2024) 217:173–82. doi: 10.1093/cei/uxae033 38616555 PMC11239561

[7] West N Chapple I Culshaw S Donos N Needleman I Suvan J . BSP implementation of prevention and treatment of peri-implant diseases - the EFP S3 level clinical practice guideline. J Dent. (2024) 149:104980. doi: 10.1016/j.jdent.2024.104980 38697506

[8] Shafizadeh M Amid R Mahmoum M Kadkhodazadeh M . Histopathological characterization of peri-implant diseases: a systematic review and meta-analysis. Arch Oral Biol. (2021) 132:105288. doi: 10.1016/j.archoralbio.2021.105288 34688133

[9] Hajishengallis G Chavakis T . Local and systemic mechanisms linking periodontal disease and inflammatory comorbidities. Nat Rev Immunol. (2021) 21:426–40. doi: 10.1038/s41577-020-00488-6 33510490 PMC7841384

[10] Hajishengallis G . Interconnection of periodontal disease and comorbidities: evidence, mechanisms, and implications. Periodontol 2000. (2022) 89:9–18. doi: 10.1111/prd.12430 35244969 PMC9018559

[11] Aoki T Hiura F Li A Yang N Takakura-Hino N Mukai S . Inhibition of non-canonical NF-κB signaling suppresses periodontal inflammation and bone loss. Front Immunol. (2023) 14:1179007. doi: 10.3389/fimmu.2023.1179007 37143646 PMC10151688

[12] Wielento A Bereta GP Łagosz-Ćwik KB Eick S Lamont RJ Grabiec AM . TLR2 activation by Porphyromonas gingivalis requires both PPAD activity and fimbriae. Front Immunol. (2022) 13:823685. doi: 10.3389/fimmu.2022.823685 35432342 PMC9010743

[13] Lobognon VD Alard JE . Could AMPs and B-cells be the missing link in understanding periodontitis? Front Immunol. (2022) 13:887147. doi: 10.3389/fimmu.2022.887147 36211356 PMC9532695

[14] Carcuac O Berglundh T . Composition of human peri-implantitis and periodontitis lesions. J Dent Res. (2014) 93:1083–8. doi: 10.1177/0022034514551754 25261052 PMC4293768

[15] Chen L Tong Z Luo H Qu Y Gu X Si M . Titanium particles in peri-implantitis: distribution, pathogenesis and prospects. Int J Oral Sci. (2023) 15:49. doi: 10.1038/s41368-023-00256-x 37996420 PMC10667540

[16] Rakic M Radunovic M Petkovic-Curcin A Tatic Z Basta-Jovanovic G Sanz M . Study on the immunopathological effect of titanium particles in peri-implantitis granulation tissue: a case-control study. Clin Oral Implants Res. (2022) 33:656–66. doi: 10.1111/clr.13928 35344630 PMC9321593

[17] Oh JM Kim Y Son H Kim YH Kim HJ . Comparative transcriptome analysis of periodontitis and peri-implantitis in human subjects. J Periodontol. (2024) 95:337–49. doi: 10.1002/jper.23-0289 37789641

[18] Oh JM Kim Y Lee HS Son H Heo HJ Baek SE . Paired transcriptional analysis of periodontitis and peri-implantitis within same host: a pilot study. J Dent. (2024) 151:105366. doi: 10.1016/j.jdent.2024.105366 39357620

[19] Chen Y Wang H Yang Q Zhao W Chen Y Ni Q . Single-cell RNA landscape of the osteoimmunology microenvironment in periodontitis. Theranostics. (2022) 12:1074–96. doi: 10.7150/thno.65694 35154475 PMC8771561

[20] Qian SJ Huang QR Chen RY Mo JJ Zhou LY Zhao Y . Single-cell RNA sequencing identifies new inflammation-promoting cell subsets in Asian patients with chronic periodontitis. Front Immunol. (2021) 12:711337. doi: 10.3389/fimmu.2021.711337 34566966 PMC8455889

[21] Jew B Alvarez M Rahmani E Miao Z Ko A Garske KM . Accurate estimation of cell composition in bulk expression through robust integration of single-cell information. Nat Commun. (2020) 11:1971. doi: 10.1038/s41467-020-15816-6 32332754 PMC7181686

[22] Newman AM Steen CB Liu CL Gentles AJ Chaudhuri AA Scherer F . Determining cell type abundance and expression from bulk tissues with digital cytometry. Nat Biotechnol. (2019) 37:773–82. doi: 10.1038/s41587-019-0114-2 31061481 PMC6610714

[23] Jin S Guerrero-Juarez CF Zhang L Chang I Ramos R Kuan CH . Inference and analysis of cell-cell communication using CellChat. Nat Commun. (2021) 12:1088. doi: 10.1038/s41467-021-21246-9 33597522 PMC7889871

[24] Jin S Plikus MV Nie Q . CellChat for systematic analysis of cell-cell communication from single-cell transcriptomics. Nat Protoc. (2025) 20:180–219. doi: 10.1101/2023.11.05.565674 39289562

[25] Lundmark A Gerasimcik N Båge T Jemt A Mollbrink A Salmén F . Gene expression profiling of periodontitis-affected gingival tissue by spatial transcriptomics. Sci Rep. (2018) 8:9370. doi: 10.1038/s41598-018-27627-3 29921943 PMC6008462

[26] Shen Z Zhang R Huang Y Chen J Yu M Li C . The spatial transcriptomic landscape of human gingiva in health and periodontitis. Sci China Life Sci. (2024) 67:720–32. doi: 10.1007/s11427-023-2467-1 38172357

[27] Schwarz F Derks J Monje A Wang HL . Peri-implantitis. J Clin Periodontol. (2018) 45 Suppl 20:S246–66. doi: 10.1007/s40496-020-00278-y 29926484

[28] Berglundh T Armitage G Araujo MG Avila-Ortiz G Blanco J Camargo PM . Peri-implant diseases and conditions: consensus report of workgroup 4 of the 2017 World Workshop on the Classification of Periodontal and Peri-Implant Diseases and Conditions. J Clin Periodontol. (2018) 45 Suppl 20:S286–91. doi: 10.1111/jcpe.12957 29926491

[29] Neurath N Kesting M . Cytokines in gingivitis and periodontitis: from pathogenesis to therapeutic targets. Front Immunol. (2024) 15:1435054. doi: 10.3389/fimmu.2024.1435054 39253090 PMC11381234

[30] Hajishengallis G Chavakis T Lambris JD . Current understanding of periodontal disease pathogenesis and targets for host-modulation therapy. Periodontol 2000. (2020) 84:14–34. doi: 10.1111/prd.12331 32844416 PMC7457922

[31] Abe T AlSarhan M Benakanakere MR Maekawa T Kinane DF Cancro MP . The B cell-stimulatory cytokines BLyS and APRIL are elevated in human periodontitis and are required for B cell-dependent bone loss in experimental murine periodontitis. J Immunol. (2015) 195:1427–35. doi: 10.4049/jimmunol.1500496 26150532 PMC4530049

[32] Newman AM Liu CL Green MR Gentles AJ Feng W Xu Y . Robust enumeration of cell subsets from tissue expression profiles. Nat Methods. (2015) 12:453–7. doi: 10.1038/nmeth.3337 25822800 PMC4739640

[33] Liu B Li F Wang Y Gao X Li Y Wang Y . APP-CD74 axis mediates endothelial cell-macrophage communication to promote kidney injury and fibrosis. Front Pharmacol. (2024) 15:1437113. doi: 10.3389/fphar.2024.1437113 39351084 PMC11439715

[34] Ma C Chen J Ji J Zheng Y Liu Y Wang J . Therapeutic modulation of APP-CD74 axis can activate phagocytosis of TAMs in GBM. Biochim Biophys Acta Mol Basis Dis. (2024) 1870:167449. doi: 10.1016/j.bbadis.2024.167449 39111632

[35] Starlets D Gore Y Binsky I Haran M Harpaz N Shvidel L . Cell-surface CD74 initiates a signaling cascade leading to cell proliferation and survival. Blood. (2006) 107:4807–16. doi: 10.1182/blood-2005-11-4334 16484589

[36] Leng L Metz CN Fang Y Xu J Donnelly S Baugh J . MIF signal transduction initiated by binding to CD74. J Exp Med. (2003) 197:1467–76. doi: 10.1084/jem.20030286 12782713 PMC2193907

[37] Shi X Leng L Wang T Wang W Du X Li J . CD44 is the signaling component of the macrophage migration inhibitory factor-CD74 receptor complex. Immunity. (2006) 25:595–606. doi: 10.1016/j.immuni.2006.08.020 17045821 PMC3707630

[38] Gore Y Starlets D Maharshak N Becker-Herman S Kaneyuki U Leng L . Macrophage migration inhibitory factor induces B cell survival by activation of a CD74-CD44 receptor complex. J Biol Chem. (2008) 283:2784–92. doi: 10.1074/jbc.m703265200 18056708

[39] David K Friedlander G Pellegrino B Radomir L Lewinsky H Leng L . CD74 as a regulator of transcription in normal B cells. Cell Rep. (2022) 41:111572. doi: 10.1016/j.celrep.2022.111572 36323260

[40] Tachida Y Miura S Muto Y Takuwa H Sahara N Shindo A . Endothelial expression of human amyloid precursor protein leads to amyloid β in the blood and induces cerebral amyloid angiopathy in knock-in mice. J Biol Chem. (2022) 298:101880. doi: 10.1016/j.jbc.2022.101880 35367207 PMC9144051

[41] Austin SA Sens MA Combs CK . Amyloid precursor protein mediates a tyrosine kinase-dependent activation response in endothelial cells. J Neurosci. (2009) 29:14451–62. doi: 10.1523/jneurosci.3107-09.2009 19923279 PMC2820274

[42] Wichienrat W Surisaeng T Sa-Ard-Iam N Chanamuangkon T Mahanonda R Wisitrasameewong W . Alveolar bone loss in a ligature-induced periodontitis model in rat using different ligature sizes. Eur J Dent. (2024) 18:933–41. doi: 10.1055/s-0044-1779426 38442914 PMC11290929

[43] Bosshardt DD . The periodontal pocket: pathogenesis, histopathology and consequences. Periodontol 2000. (2018) 76:43–50. doi: 10.1111/prd.12153 29194796

[44] Gao J Cai S Wang Z Li D Ou M Zhang X . The optimization of ligature/bone defect-induced periodontitis model in rats. Odontology. (2022) 110:697–709. doi: 10.21203/rs.3.rs-880589/v1 35654915

[45] Ali M Yang F Plachokova AS Jansen JA Walboomers XF . Application of specialized pro-resolving mediators in periodontitis and peri-implantitis: a review. Eur J Oral Sci. (2021) 129:e12759. doi: 10.1111/eos.12759 33565133 PMC7986752

[46] Lin P Niimi H Ohsugi Y Tsuchiya Y Shimohira T Komatsu K . Application of ligature-induced periodontitis in mice to explore the molecular mechanism of periodontal disease. Int J Mol Sci. (2021) 22:8900. doi: 10.3390/ijms22168900 34445604 PMC8396362

[47] Zhong M Huang J Wu Z Chan KG Wang L Li J . Potential roles of selectins in periodontal diseases and associated systemic diseases: could they be targets for immunotherapy? Int J Mol Sci. (2022) 23:14280. doi: 10.3390/ijms232214280 36430760 PMC9698067

[48] Komatsu T Nagano K Sugiura S Hagiwara M Tanigawa N Abiko Y . E-selectin mediates Porphyromonas gingivalis adherence to human endothelial cells. Infect Immun. (2012) 80:2570–6. doi: 10.1128/iai.06098-11 22508864 PMC3416463

[49] Muller WA Weigl SA Deng X Phillips DM . PECAM-1 is required for transendothelial migration of leukocytes. J Exp Med. (1993) 178:449–60. doi: 10.1002/3527604669.ch13 8340753 PMC2191108

[50] Woodfin A Voisin MB Nourshargh S . PECAM-1: a multi-functional molecule in inflammation and vascular biology. Arterioscler Thromb Vasc Biol. (2007) 27:2514–23. doi: 10.1161/atvbaha.107.151456 17872453

[51] Chan WK Williams J Sorathia K Pray B Abusaleh K Bian Z . A novel CAR-T cell product targeting CD74 is an effective therapeutic approach in preclinical mantle cell lymphoma models. Exp Hematol Oncol. (2023) 12:79. doi: 10.1186/s40164-023-00437-8 37740214 PMC10517521

[52] Camacho Meza G Avalos Navarro G De La Cruz Mosso U Ramírez Patiño R Muñoz Valle JF Bautista Herrera LA . Macrophage migration inhibitory factor: Exploring physiological roles and comparing health benefits against oncogenic and autoimmune risks (Review). Int J Mol Med. (2025) 56:149. doi: 10.3892/ijmm.2025.5590 40682854 PMC12306601

[53] Balta MG Papathanasiou E Blix IJ Van Dyke TE . Host modulation and treatment of periodontal disease. J Dent Res. (2021) 100:798–809. doi: 10.1177/0022034521995157 33655803 PMC8261853

