# Supplementary material for: Multi-omics comparison of periodontitis and peri-implantitis identifies plasma cell enrichment as a shared feature and a periodontitis-associated endothelial–plasma cell APP–CD74 axis
Source: Front Immunol. 2026 Jul 2;17:1867656. doi: 10.3389/fimmu.2026.1867656 (PMC13372705; doi:10.3389/fimmu.2026.1867656)
Supplement: Supplementary file 1 [file DataSheet1.docx]

**Supplementary Figure Legends**

**Supplementary Figure 1. Functional enrichment analysis of PD-specific and PI-specific validated differentially expressed genes.**

**(A)** Venn diagram showing the cross-dataset validation of PD-specific DEGs. The intersection (n = 820) represents genes consistently dysregulated in PD vs. healthy across both GSE223924 and GSE106090, exclusive of PI-overlapping DEGs. **(B)** Bar chart of GO enrichment (top 5 terms per category) for PD-specific genes. Bars are colored by GO category: Biological Process (BP, red), Cellular Component (CC, teal), and Molecular Function (MF, blue). **(C)** Top 15 Reactome pathways enriched in PD-specific genes. **(D)** Top 15 KEGG pathways enriched in PD-specific genes. In (C–D), bar color reflects −log₁₀(P-value). **(E)** Venn diagram showing cross-dataset validation of PI-specific DEGs (n = 1,832 shared genes). **(F)** GO enrichment bar chart (top 5 per category) for PI-specific genes, using the same color scheme as (B). **(G)** Top 15 Reactome pathways enriched in PI-specific genes. **(H)** Top 15 KEGG pathways enriched in PI-specific genes. DEGs, differentially expressed genes; PI, peri-implantitis; PD, periodontitis; BP, biological process; CC, cellular component; MF, molecular function.

**Supplementary Figure 2. Violin plots of significantly altered cell populations identified by BisqueRNA deconvolution.**

**(A)** GSE106090: violin plots for the five cell populations showing significant group differences, including cDC1, Endothelial Cells, Neutrophils, Pericytes, and Plasma Cells. **(B)** GSE223924: violin plots for the eight significantly altered cell populations, including CD4+ T Cells, CD8+ T Cells, Endothelial Cells, Epithelial Cells, Fibroblasts, Macrophages, Mast Cells, and Plasma Cells. Each panel shows violin plots with embedded box plots and individual data points. Pairwise comparisons were performed using the Wilcoxon rank-sum test with BH correction. *, FDR < 0.05; **, FDR < 0.01; ***, FDR < 0.001; ns, not significant.

**Supplementary Figure 3. Violin plots of significantly altered immune cell populations identified by CIBERSORTx.**

**(A)** GSE106090: violin plots for the seven significantly altered immune populations, including Plasma cells, resting and activated CD4 memory T cells, T follicular helper cells, activated NK cells, M1 Macrophages, and resting Dendritic cells. **(B)** GSE223924: violin plots for the eight significantly altered immune populations, including Plasma cells, T follicular helper cells, M0 and M2 Macrophages, resting Dendritic cells, resting and activated Mast cells, and Neutrophils. Each panel shows violin plots with embedded box plots and individual data points. Pairwise comparisons were performed using the Wilcoxon rank-sum test with BH correction. *, FDR < 0.05; **, FDR < 0.01; ***, FDR < 0.001; ns, not significant.

**Supplementary Figure 4. Plasma cell–centered intercellular communication network in healthy gingival tissue.**

**(A)** Circle plot showing outgoing communication from Plasma Cells to other cell populations in the healthy condition. **(B)** Circle plot showing incoming communication to Plasma Cells from other cell populations in the healthy condition. **(C)** Bubble plot showing ligand-receptor interactions in which Plasma Cells act as receivers in health. The x-axis indicates source cell populations. **(D)** Bubble plot showing ligand-receptor interactions in which Plasma Cells act as senders in health. The x-axis indicates target cell populations. Dot color represents communication probability, and dot size indicates statistical significance. Comparison with Figure 5 highlights disease-associated remodeling of the plasma cell communication network, particularly the emergence of strong endothelial inputs to Plasma Cells and gain of endothelial adhesion-related outgoing signaling in periodontitis.

**Supplementary Figure 5. Targeted gene expression and deconvolution-based validation of the plasma cell-endothelial program.**

**(A)** Targeted expression analysis of hub genes, plasma cell markers, plasma cell-associated receptor genes, endothelial markers, and endothelial-associated ligand or matrix genes in GSE106090 across healthy, PD, and PI groups. **(B)** Targeted expression analysis of the same gene panel in GSE223924 across healthy, PD, and PI groups. **(C)** Independent deconvolution validation in GSE10334 and GSE16134 using CIBERSORTx and BisqueRNA. **(D)** Global CellChat network visualization in GSE164241 showing broad intercellular communication patterns in healthy and periodontitis samples. Edge thickness represents overall communication strength. PD, periodontitis; PI, peri-implantitis.
