# Supplementary figures and images for: Multi-omics comparison of periodontitis and peri-implantitis identifies plasma cell enrichment as a shared feature and a periodontitis-associated endothelial–plasma cell APP–CD74 axis
Source: Front Immunol. 2026 Jul 2;17:1867656. doi: 10.3389/fimmu.2026.1867656 (PMC13372705; doi:10.3389/fimmu.2026.1867656)

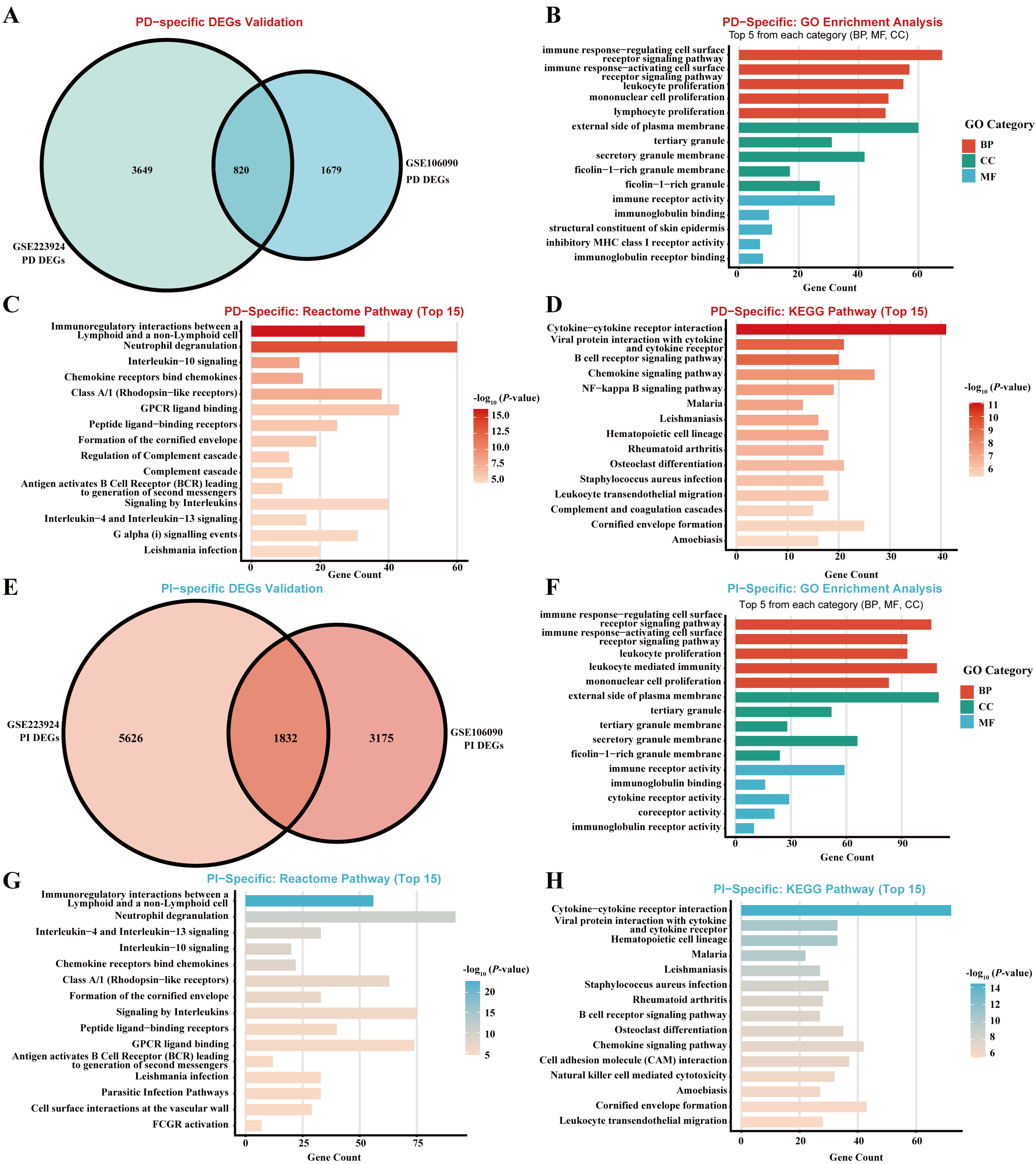

Supplement: Supplementary file 2 [file Image1.tif]

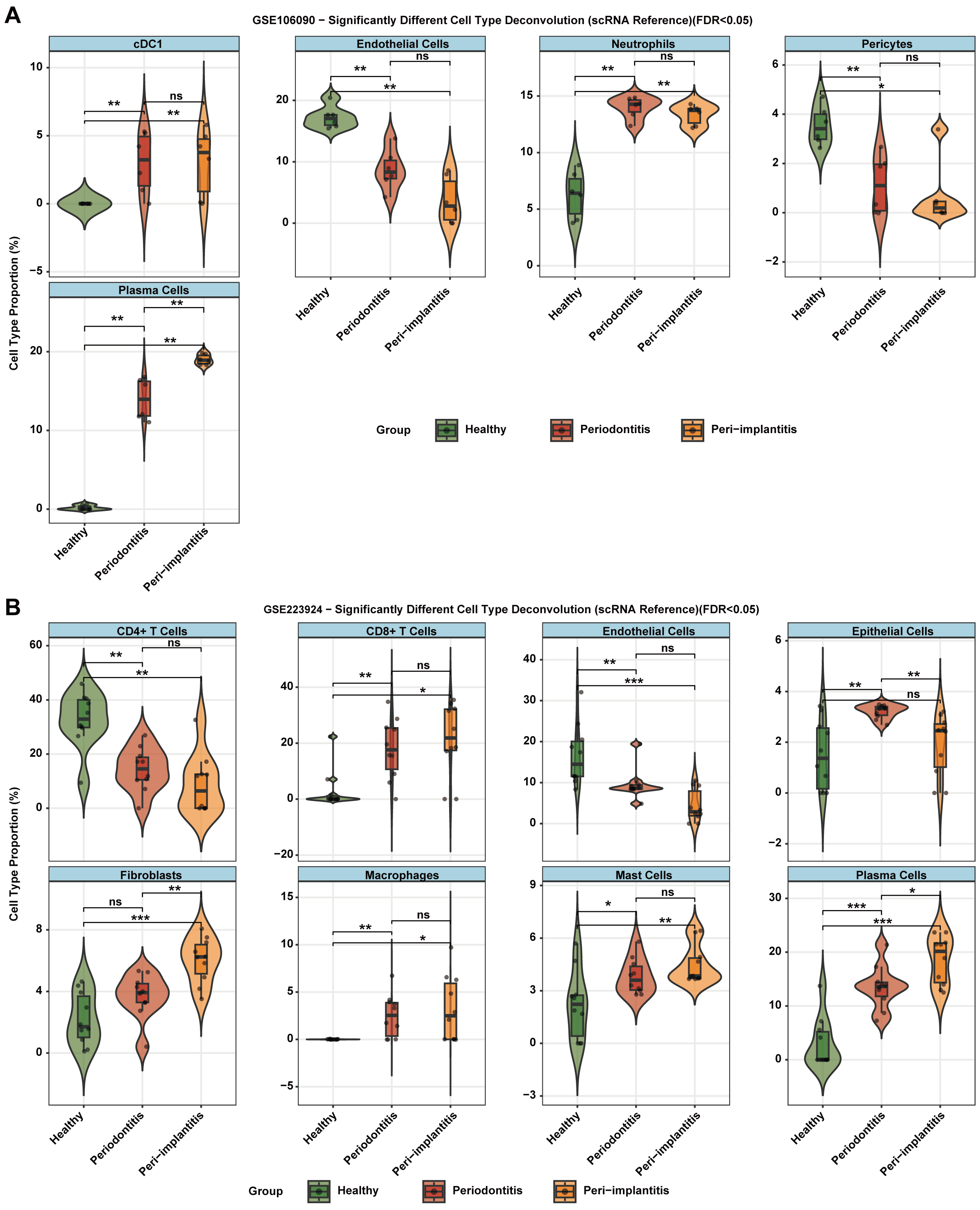

Supplement: Supplementary file 3 [file Image2.tif]

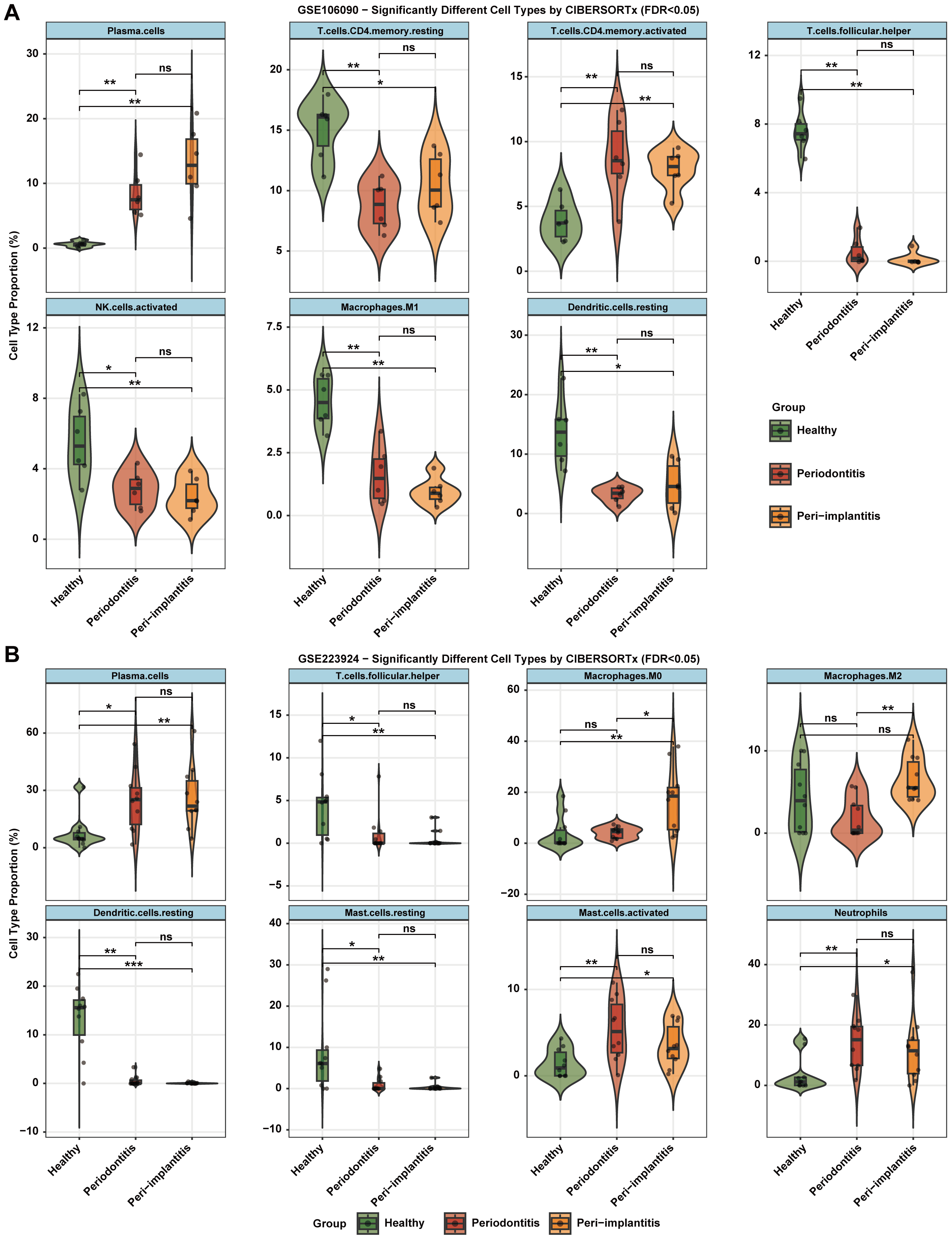

Supplement: Supplementary file 4 [file Image3.tif]

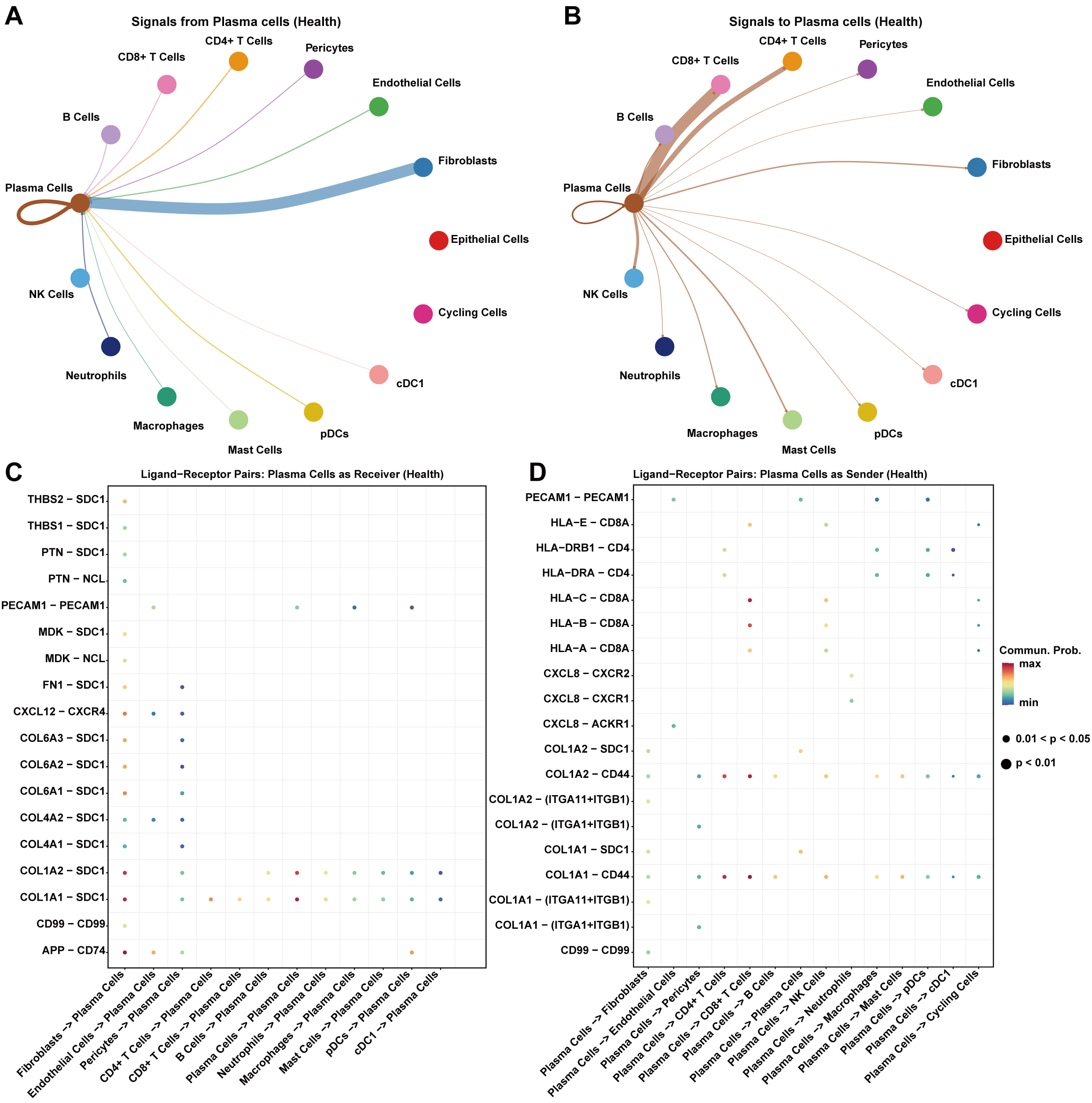

Supplement: Supplementary file 5 [file Image4.tif]

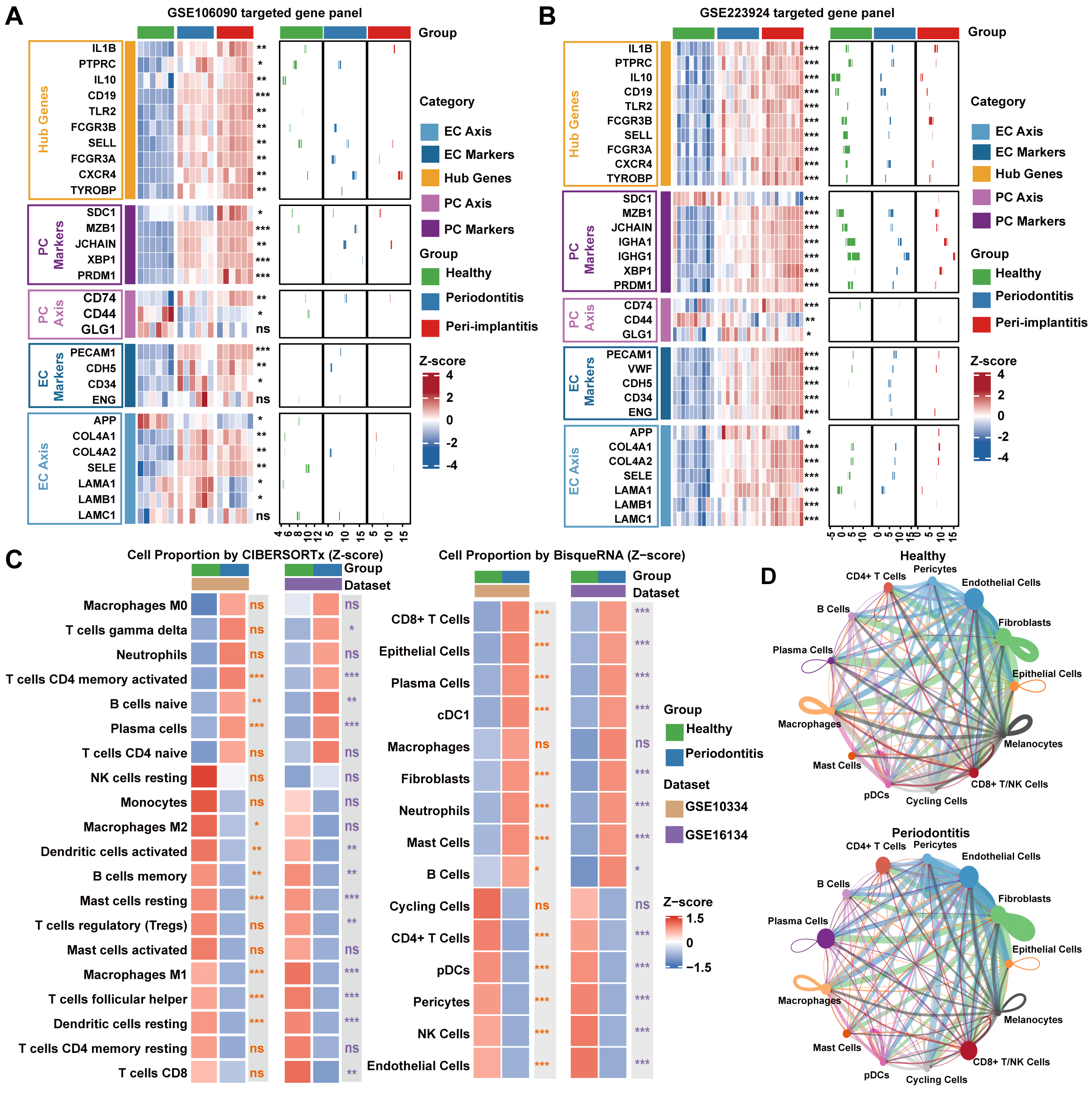

Supplement: Supplementary file 6 [file Image5.tif]
